# The Glutamatergic System Regulates Feather Pecking Behaviors in Laying Hens Through the Gut–Brain Axis

**DOI:** 10.3390/ani15091297

**Published:** 2025-04-30

**Authors:** Xiliang Yan, Chao Wang, Yaling Li, Yating Lin, Yinbao Wu, Yan Wang

**Affiliations:** 1 Heyuan Branch, Guangdong Laboratory for Lingnan Modern Agriculture, College of Animal Science, South China Agricultural University, Guangzhou 510642, China; yanxiliang1991@163.com (X.Y.); 13355502761@163.com (C.W.); 18241674143@163.com (Y.L.); 19875216900@163.com (Y.L.); wuyinbao@scau.edu.cn (Y.W.); 2 Guangdong Provincial Key Lab of Agro-Animal Genomics and Molecular Breeding, South China Agricultural University, Guangzhou 510642, China; 3 National Engineering Research Center for Breeding Swine Industry, College of Animal Science, South China Agricultural University, Guangzhou 510642, China

**Keywords:** feather pecking, glutamatergic system, gut–brain axis, intestinal microbiota, animal welfare

## Abstract

Feather pecking (FP) is a significant welfare and economic problem in laying hen husbandry. While there is growing evidence that the glutamatergic system plays a crucial role in regulating FP behavior, the biological mechanisms remain unclear, largely due to the limited uptake of peripheral glutamate across the blood–brain barrier (BBB). Here, we applied a multi-omics approach combined with physiology assays to answer this question from the perspective of the gut–brain axis. A total of 108 hens were randomly assigned to two groups (treatment and control) with six replicates each, and the treatment group was subjected to chronic environmental stressors including re-housing, noise, and transport. We found that chronic exposure to environmental stressors induced severe FP, accompanied by reduced production performance and increased anxiety- and depression-related behaviors, compared to controls. In addition, the immune system was potentially disrupted in FP chickens. Notably, gut microbiota diversity and composition were significantly altered, leading to decreased microbial community stability. Non-targeted metabolomic analysis identified a variety of differential metabolites, primarily associated with arginine and histidine biosynthesis. A significant increase in glutamate levels was also observed in the hippocampus of FP chickens. Transcriptome analysis revealed the upregulated expressions of glutamate-related receptors *GRIN2A* and *SLC17A6* in the hippocampus. Correlation analysis indicated that *GRIN2A* and *SLC17A6* are positively associated with arginine levels in the duodenum, while *Romboutsia* in the duodenum is negatively correlated with arginine. These findings suggest that intestinal bacteria, including *Romboutsia*, may influence FP behavior by altering plasma arginine and histidine levels. These changes, in turn, affect glutamate levels and receptor gene expression in the hippocampus, thereby regulating the glutamatergic system. Our research offers insights into novel strategies for mitigating harmful behaviors in poultry farming, with potential benefits for animal performance and welfare.

## Simple Summary

This study investigates the biological mechanism of harmful FP behavior in laying hens induced by chronic stress. We found that gut microbes like *Romboutsia* may increase the plasma arginine and histidine levels by enhancing their biosynthesis and suppressing catabolic pathways, thereby elevating glutamate levels and *GRIN2A* and *SLC17A6* gene expression in the hippocampus. These neurochemical shifts ultimately regulate the glutamatergic system to influence the FP behavior in laying hens. These findings highlight the gut–brain axis as a critical regulator of FP, offering scientific support to develop targeted strategies to mitigate FP behavior.

## 1. Introduction

For centuries, laying hens have been used to provide a protein source for humanity. In China, the total population of laying hens in 2022 was approximately 946 million (data from http://www.moa.gov.cn/ (accessed on 11 May 2023)), making the poultry industry one of the most large-scale, intensive, and industrialized agricultural sectors. FP is an abnormal and harmful behavior that often occurs in intensive farming of laying hens [1]. It may occur among birds of any age or any breed and can lead to skin and tissue damage, increasing the risk of infections [2]. In addition, FP increases the heat loss and feed intake of laying hens, thus negatively affecting their egg production efficiency. While pecking behavior can be effectively reduced through various measures, like beak trimming, these methods raise significant welfare concerns due to stress and pain in birds, and a loss of beak sensation and function. Therefore, understanding the biological mechanism of pecking behavior can develop more effective humane mitigating strategies [3].

Although FP has been extensively studied, its underlying biological mechanisms remain incompletely understood [4,5]. Several studies have linked FP to multiple neurotransmitters in the central nervous system [6,7,8,9], including serotonin (5-hydroxytryptamine, 5-HT), dopamine (DA), γ-aminobutyric acid (GABA), and glutamate. Glutamate, the most prevalent neurotransmitter in the brain and spinal cord, is involved in at least 90% of excitatory synapses and regulates numerous brain functions, including learning, memory, and cognition. In the hippocampus of chickens displaying intense FP, glutamate levels were found to be 1.51 times higher than in control chickens, and with significantly increased levels of its precursor, histidine [9]. The upregulated expression of genes (*GRIN1*, *GRIN2A*, and *GRIN2B*) encoding the voltage-sensitive ionotropic glutamate receptor (N-Methyl-D-Aspartic acid, NMDA) has also been observed in pecking chickens [10]. However, the precise role of the glutamatergic system in FP remains unclear due to the BBB’s limited permeability to peripheral glutamate.

As a virtual endocrine organ, the gut microbiota can influence host behavior by regulating glutamatergic, GABA receptor, and the corresponding gene expression. Studies have shown that gut microbiota from patients with schizophrenia can alter the glutamate–glutamine–GABA cycle and induce schizophrenia-relevant behaviors in mice [11]. Similarly, microbiota transplantation from depressed patients can alter arginine, proline, and histidine metabolism, leading to emotionally impaired phenotypes in mice [12]. Gut microbiota can also synthesize various neurotransmitters and modulate gene expressions in the central nervous system, influencing a range of host behaviors. For example, *Lactobacillus rhamnosus* can produce GABA neurotransmitters and reduce depression and anxiety-like behavior in mice [13]. Our previous research revealed significantly higher glutamate levels in the hippocampus of FP laying hens, along with significant alterations in the cecal bacterial community [9]. Based on these findings, we hypothesized that gut microbiota may influence the central glutamatergic system by altering the metabolism of histidine and other glutamate precursor substances.

## 2. Materials and Methods

### 2.1. Experimental Instruments and Biochemical Kits

Details of experimental instruments and biochemical kits can be found in Tables S1 and S2.

### 2.2. Animals and Housing Conditions

A total of 108 healthy, 27-week-old Hyland Gray laying hens with similar egg production rates and body weights were purchased from a large-scale commercial market in Maoming City, Guangdong Province, China. The chickens were randomly assigned to two experimental groups (*n* = 54 per group), each consisting of six replicates with 9 chickens per replicate (cage). Birds were housed in a closed single level cage (1.5 m × 0.6 m × 0.5 m) under conventional commercial farm management conditions. During the experiment, artificial lighting was provided from 6:00 a.m. to 10:00 p.m., with light intensity maintained at 12–14 lx, and the temperature inside the house was kept at 25 ± 4 °C. The treatment group was exposed to chronic and unpredictable stressors, while the control group was raised under standard conditions. A numbered silicone backpack (8 cm × 6 cm × 0.5 cm) affixed to each chicken’s back served as an individual identifier.

### 2.3. Stress Treatments

**Figure 1:**
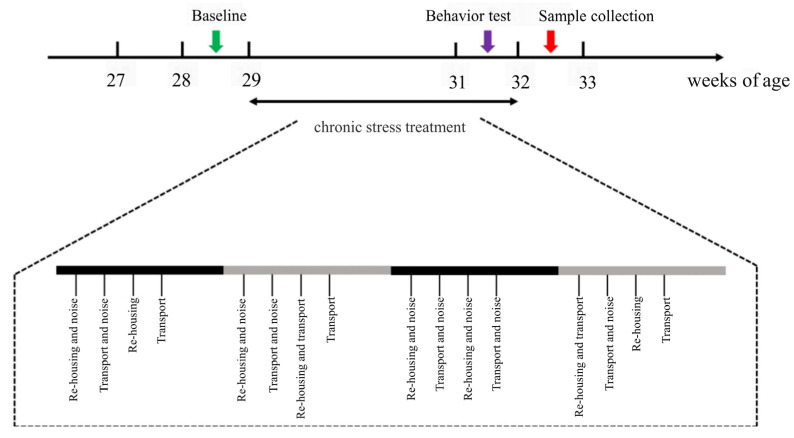
A schematic diagram designed for chronic stress treatment. During Weeks 29–32, the treatment group of laying hens were subjected to chronic and unpredicted stressors including re-housing, noise, and transport. Behavior assessments during Week 28 were assigned as the baseline of FP. The open field (OF) test was performed at Week 31, and blood and tissue samples were collected for further analysis after stress treatments.

The experimental timeline and stress treatment workflow are depicted in Figure 1. A two-week pre-feeding period (Weeks 27 and 28) allowed all birds to acclimate to the environment. During Week 28, baseline FP behavior was assessed over four days. Beginning at 29 weeks of age, the treatment group was exposed to four weeks of chronic stressors, consisting of re-housing, noise, and transport. Re-housing involved dividing the chickens within each cage into two subgroups (4 or 5 chickens per subgroup) and mixing them with subgroups from different cages. The noise stressor consisted of 100 dB sound pressure at frequencies between 605 and 3112 Hz, delivered in four second bursts with three replicates every 2–10 min. Transport stressor involved moving each cage back and forth for two minutes.

### 2.4. Behavioral Assessments

During the stress treatment period (Weeks 29–32), behavioral observations were recorded five times per week for 20 min (10 min at 9:00 a.m. and 10 min at 3:00 p.m.). FP behavior was defined as continuous pecks directed toward the same body part of the same chicken, and categorized as severe FP or gentle FP. Gentle FP was further subdivided into exploratory FP and stereotyped FP. Details about the different types of FP can be found in Table S3. For each observation period, the initiator and receiver of FP interactions, the number of pecks, and the type of peck were recorded. A “feather pecker” was defined as a chicken initiating an aggressive peck, a “feather victim” as a chicken receiving an aggressive peck, and a “neutral chicken” as a chicken neither initiating nor receiving aggressive pecks. Two trained observers conducted all behavioral observations.

### 2.5. Sample Collection

At 32 weeks of age, 24 chickens were selected for biological sample collection: 12 exhibiting the most severe FP behavior and 12 neutral chickens from the control group. Blood samples were collected from the brachial wing veins, placed on ice for one hour, and centrifuged (860× *g*, 4 °C, 10 min) to prepare plasma. Following plasma collection, the selected chickens were euthanized by cervical dislocation. Ileal, cecal, and duodenal contents were collected. The brain was rapidly dissected, and the hippocampus and amygdala were collected. Plasma, brain tissue, and intestinal contents were immediately transferred to cryotubes and stored at −80 °C for subsequent analysis.

### 2.6. Quantification of Immune Response Markers

Enzyme-linked immunosorbent assay (ELISA) kits were used to measure immune response markers, including immunoglobulin A (IgA), immunoglobulin G (IgG), immunoglobulin M (IgM), interleukin 1 (IL-1), interleukin 6 (IL-6), tumor necrosis factor α (TNF-α), corticosterone (CORT), epinephrine (EPI), and norepinephrine (NE). Optical density was measured at 450 nm using Nessler’s reagent spectrophotometry (Thermo, MA, America). As an advanced immunological technique, the ELISA kits were commonly used to measure proteins, antibodies, antigens, and hormones in biological samples, including chicken plasma. Examples include the determination of cytokine levels (CORT and IL-1β) [14] and immune parameters (IgG and IgM) [15] in chicken serum.

### 2.7. The 16S rRNA Gene Quantification

The 16S rRNA gene quantification comprised three steps: DNA isolation, library preparation, and sequencing. Microbial DNA was extracted from duodenal, ileal, and cecal samples using the QIAamp PowerFecal DNA Kit (QIAGEN, Hilden, Germany), following the manufacturer’s protocol. DNA quality was assessed by measuring purity and concentration using an ultra-micro spectrophotometer. DNA was diluted to 1 ng/μL. Using diluted DNA as a template, the 16S rRNA gene was amplified with barcoded primers (341F: 5′-CCTAYGGGRBGCASCAG-3′; 806R: 5′-GGACTACNNGGGTATCTAAT-3′) flanking the V3-V4 hypervariable regions. PCR products were assessed by 2% agarose gel electrophoresis and pooled in equal proportions based on PCR concentrations. DNA libraries were constructed using the NEBNext^®^ UltraTM II DNA Library Prep Kit (Illumina, San Diego, CA, USA) and quantified by Qubit and Q-PCR. The final library was sequenced on a NovaSeq6000 system (Illumina, San Diego, CA, USA). PCR amplification and 16S rRNA gene sequencing were performed by Beijing Novel Biosciences Co., Ltd. (Beijing, China).

### 2.8. Eukaryotic Transcriptome Sequencing

Total RNA was purified from the hippocampus and amygdala tissues using the RNAprep Pure Tissue Kit (TIANGEN, Beijing, China). RNA concentration was measured using an ultra-micro spectrophotometer, and samples with a concentration > 100 ng/μL, OD260/280 ≥ 1.5, and OD260/230 ≥ 1.5 were retained. rRNA was removed using the Ribo-zero kit (Illumina, San Diego, CA, USA). Libraries were generated using the Illumina TruseqTM RNA sample prep Kit (Illumina, San Diego, CA, USA). PolyA-tailed eukaryotic mRNA was enriched, fragmented by sonication, and used as a template for first-strand cDNA synthesis with M-MuLV reverse transcriptase. The RNA strand was degraded with RNase H, followed by second-strand cDNA synthesis with DNA polymerase I using dNTPs as substrate. Double-stranded cDNA was ligated to adapter sequences. The cDNA was amplified by PCR and purified with AMPure XP beads (Beckman Coulter, Brea, CA, USA). The final products were sequenced and analyzed by Shanghai BioZero Biotech Co., Ltd. (Shanghai, China).

### 2.9. Statistical Analysis

SPSS 26 was used for independent samples *t*-test. Statistical significance was defined as *p* < 0.05. Data are presented as mean ± SE (standard error). GraphPad Prism 8.0 was used for data visualization (FP behavior, production performance, egg quality, immune and stress response markers). The processing details of omics data are described in Methods S1–S3. Correlation analysis and heatmap generation were performed using R (Version 4.1.1). Network visualization and analysis were performed using Gephi 0.10.1 and Cytoscape 3.8.0.

## 3. Results

### 3.1. Stress-Induced FP Initiates a Cascade of Adverse Physiological and Behavioral Changes in Laying Hens

**Figure 2:**
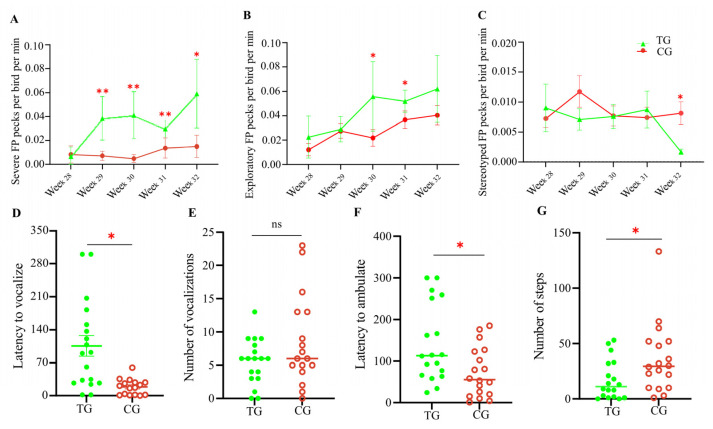
Stress-induced behavior changes in laying hens. The pecking frequency of severe FP (**A**), exploratory FP (**B**), and stereotyped FP (**C**) behaviors between two groups of laying hens during four-week stress treatments are shown. The behaviors of laying hens in OF tests in terms of vocalization (**D**,**E**) and ambulating (**F**,**G**). TG, treatment group; CG, control group. * *p* < 0.05; ** *p* < 0.01. Each group in the pecking frequency test consisted of six chickens; each group in the OF test consisted of eighteen chickens. ns means no statistical difference.

As expected, there was no significant difference in the frequency of severe FP or gentle FP behaviors between the two groups of 28-week-old laying hens before stress treatment began. However, the two groups exhibited distinct FP behaviors during Weeks 29–32. As shown in Figure 2A, the frequency of severe FP behaviors in the stress treatment group was significantly higher than in the control group (*p* < 0.05) and showed a steady increase over time. At Week 32, there was a four-fold difference in the number of birds exhibiting severe FP behavior between the two groups, with average threat frequencies per bird of 0.059 and 0.015 pecks/min for the stress treatment and control groups, respectively. Additionally, the stress treatment group displayed more frequent exploratory FP behaviors than the control group, particularly during Weeks 30–31 (*p* < 0.05) (Figure 2B). Interestingly, the control group tended to exhibit more stereotyped FP behaviors than the stress treatment group (Figure 2C). This form of FP typically does not result in significant feather damage and is considered normal investigatory behavior. These results indicate that stressed birds primarily exhibited severe FP behaviors, causing serious injury or even death to the recipients.

Figure 2D–G show the behavioral performance of the two groups in the open field (OF) test (Method S4). The vocalization latency of laying hens in the stress treatment group was significantly higher compared with the control group (*p* < 0.05). However, there was no significant difference in the number of vocalizations. Furthermore, the stress-treated birds took significantly longer to ambulate (*p* < 0.05) and the number of steps was significantly reduced (*p* < 0.05), indicating lower activity, compared with the control group. These results demonstrate that laying hens subjected to chronic and unpredictable stressors exhibit higher levels of fear and depression.

Production performance and egg quality are important economic traits in laying hens (Method S5). These indicators, including daily feed intake, egg weight, egg shape index, eggshell thickness, etc., are summarized in Figures S1 and S2. No significant differences were observed between the two groups in average daily feed intake, average egg weight, or feed conversion ratio. However, the egg-laying rate of the stress treatment group was significantly lower than that of the control group (*p* < 0.05), decreasing by 5.52% and 7.57% at Weeks 30 and 32, respectively. Moreover, the total egg weight of the stress treatment group also significantly decreased by 6.93% at Week 32 (*p* < 0.05). Chronic and unpredictable stress decreased egg shape index (*p* < 0.05), eggshell thickness (*p* < 0.01), and eggshell strength (*p* < 0.05). Albumen height and Haugh unit did not differ between the groups. Overall, FP behaviors adversely affected the production performance and egg quality of the laying hens.

The effect of FP behaviors on the immune system was further investigated. As shown in Table S4, the levels of proinflammatory cytokines (IL-1, IL-6, and TNF-α) and immunoglobulins (IgA, IgG, and IgM) were significantly reduced in the stress-treated laying hens at the end of the four-week experimental period (*p* < 0.05). These indicators are critical for initiating the inflammatory response and maintaining the immune system. Furthermore, a significant increase in catecholamines (EPI and NE) was observed in the stress treatment group (*p* < 0.05). Extreme levels of individual catecholamines contribute to numerous adverse effects, such as anxiety, fatigue, and depression. These results suggest that long-term stress causes a potential perturbation in the immune system of FP chickens.

### 3.2. Gut Microbiota Diversity and Composition of Laying Hens Are Altered by Stress-Induced FP

**Figure 3:**
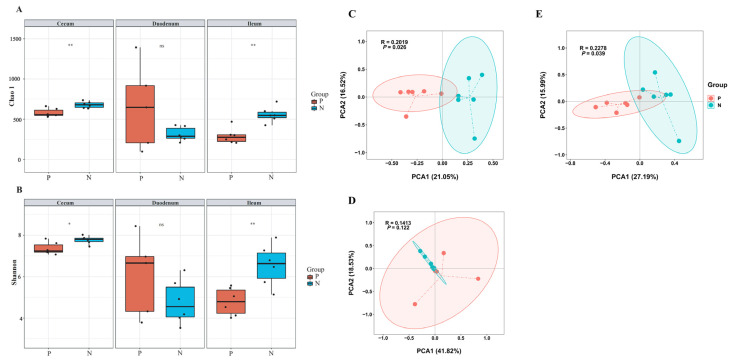
Diversity analysis of gut microbiota. The alpha diversity of gut microbiota is based on the Chao1 index (**A**) and the Shannon index (**B**). The beta diversity of gut microbiota in the Cecum (**C**), Duodenum (**D**), and Ileum (**E**) is based on principal coordinate analysis. P, pecker; N, neutral. Each group consisted of six chickens. ns means no statistical difference. * means *p* < 0.05 and ** represents *p* < 0.01.

The gut microbiota in chickens is closely related to their health status and production performance. To further understand the relationship between gut microbiota and FP in laying hens, 16S rRNA gene sequencing was used to evaluate microbiota changes between the two groups. Figure 3A,B depict the alpha diversity of the microbiota in the cecum, duodenum, and ileum. Chao1 and Shannon indices were used to quantify species richness and diversity, respectively. Species richness in the cecum and ileum of feather peckers was significantly lower than in neutral chickens (*p* < 0.05). Species diversity was also significantly reduced in the cecum and ileum of feather-pecking chickens (*p* < 0.05). The alpha diversity of gut microbiota in the duodenum did not differ significantly between the two groups. To determine whether FP was associated with altered microbiota composition, beta diversity analysis was performed. As shown in Figure 3C–E, the principal coordinate analysis (PCoA) of the microbiome beta diversity further demonstrated that cecal and ileal microbiota composition differed significantly between chickens with FP and neutral chickens (*p* < 0.05). There was no significant difference in duodenal microbiota beta diversity between the groups. These results indicate that stress-induced FP significantly alters gut microbiota diversity and composition in laying hens.

**Figure 4:**
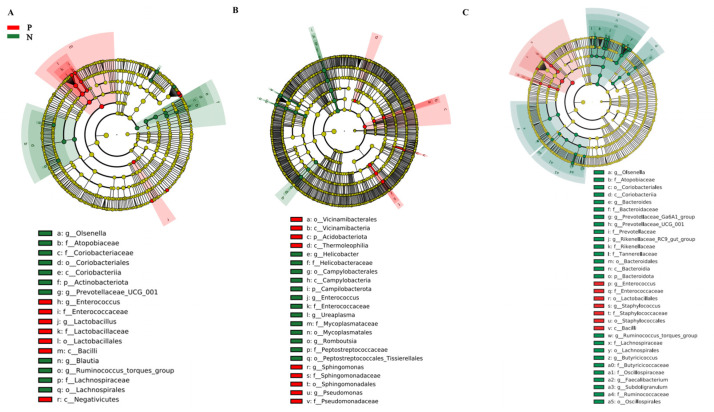
Identification of differential gut microbiota. LEfSe analysis of gut microbiota in the cecum (**A**), duodenum (**B**), and ileum (**C**). Each analysis involved six chickens.

The relative abundance of gut microbiota at the phylum level is shown in Figure S3. Three major bacterial phyla were identified in the cecum (Figure S3A): *Firmicutes* (~55.7%), *Bacteroidota* (~32.7%), and *Actinobacteriota* (~6.47%). In the duodenum (Figure S3B), the dominant phyla were *Firmicutes* (~73.3%), *Campilobacterota* (~11.9%), and *Proteobacteria* (~5.48%). In the ileum (Figure S3C), the dominant phyla were *Firmicutes* (~82.1%), *Bacteroidota* (~9.24%), and *Actinobacteriota* (~5.13%). Figure S4 shows the relative abundance of gut microbiota at the genus level. In the cecum (Figure S4A), the dominant genera were *Lactobacillus* (~23.3%), *Bacteroides* (~17.9%), and *Olsenella* (~5.71%). In the duodenum (Figure S4B), the dominant genera were *Lactobacillus* (~60.9%), *Helicobacter* (~12.2%), and *Aeriscardovia* (~3.27%). In the ileum (Figure S4C), the dominant genera were *Lactobacillus* (~52.1%), *Romboutsia* (~15.2%), and *Bacteroides* (~5.70%). Linear discriminant analysis effect size (LEfSe) was used to identify differential gut microbiota between the groups. At the genus level, *Lactobacillus* and *Enterococcus* were enriched in the cecum of FP chickens, while the *Olsenella* and *Ruminococcus torques group* were enriched in neutral chickens (Figure 4). In the duodenum, *Sphingomonas* and *Pseudomonas* characterized FP chickens, while *Helicobacter* and *Romboutsia* were identified in neutral chickens. In the ileum, *Enterococcus* and *Staphylococcus* were enriched in FP chickens, while *Bacteroides* and *Olsenella* were enriched in neutral chickens. These results demonstrate that FP is characterized by disturbed gut microbiota.

As described above, cecal and duodenal microbiota compositions differed significantly between the two groups. To investigate gut microbiota stability, Spearman’s correlation coefficient was used to visualize the co-occurrence network of core bacteria (Figure S5; |Rspearman| > 0.6, *p* < 0.05). The network structure differed distinctly between the groups. In the cecum, the FP group had 689 links and 159 nodes, while the control group had 917 links and 187 nodes. Similarly, in the duodenum, the FP group had fewer links and nodes than the control group (decreases of 259 and 16, respectively). These results indicate decreased gut microbiota stability in FP chickens. PICRUSt2 was used to predict gut microbiota functionality. PICRUSt2 analysis revealed 377, 425, and 402 predicted metabolic pathways from cecal, duodenal, and ileal microbiota gene expression, respectively (Figure S6). Compared with neutral chickens, several gene expressions related to amino acid biosynthesis were upregulated in FP chickens. For example, arginine biosynthesis II and L-histidine biosynthesis were significantly enhanced in the cecum of FP chickens. Arginine biosynthesis I and arginine biosynthesis IV were significantly activated in the duodenum. In the ileum, histidine biosynthesis, arginine biosynthesis III, the superpathway of arginine and polyamines, and other pathways were upregulated.

### 3.3. A Variety of Distinct Metabolites Are Identified in the FP Group

The gut microbiota plays a significant role in regulating host metabolism, and its disruption can lead to a wide range of metabolic diseases. Therefore, we investigated how FP behaviors perturb microbial metabolism in laying hens. Non-targeted metabolomics, a global and comprehensive analysis method, allowed us to identify a range of metabolic features in these organisms (Method S6 and Figure S7). In total, 724, 572, 599, and 389 metabolite features in positive ion mode and 303, 320, 288, and 219 in negative ion mode were extracted from peripheral blood plasma, cecum, duodenum, and ileum, respectively. Additionally, 758 and 639 metabolites were identified from hippocampus and amygdala samples, respectively. To determine metabolic differences between the two groups, orthogonal partial least squares–discriminant analysis (OPLS-DA) was performed. Metabolites were considered differential if their variable importance in projection (VIP) was not less than 1.0 and the *p*-value of the *t*-test was less than 0.05. Using these criteria, 121 and 28 differential metabolites were detected in hippocampus and amygdala samples, respectively. Of these, 101 and 48 metabolites showed higher levels in FP chickens. Univariate and multivariate analysis also revealed 147 and 81 significantly altered metabolites in the intestine and peripheral blood plasma of FP chickens, respectively. Details of these differential metabolites are provided in Table S5.

**Figure 5:**
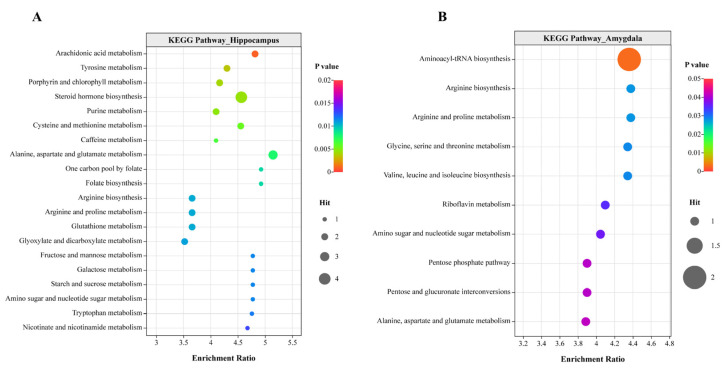
KEGG pathway enrichment analysis. Pathway enrichment of differentially accumulated metabolites in the hippocampus (**A**) and amygdala (**B**). The bubble size represents the number of metabolites; the color bar represents the corrected *p* value. Each analysis involved six chickens. Permission was obtained from Kanehisa Laboratories to use the KEGG pathway database [16].

The identified differential metabolites were mapped to KEGG metabolic pathways for functional enrichment analysis. As shown in Figure 5, a total of 20 and 10 significantly enriched metabolic pathways were identified in the hippocampus and amygdala, respectively, including “arachidonic acid metabolism”, “tyrosine metabolism”, “steroid hormone biosynthesis”, “arginine biosynthesis”, and “alanine, aspartate and glutamate metabolism”. There were 5, 11, 19, and 22 significantly enriched metabolic pathways in the cecum, duodenum, ileum, and peripheral blood plasma, respectively, related to “arginine biosynthesis”, “arachidonic acid metabolism”, “arginine”, “proline metabolism”, “pyrimidine metabolism”, and others (Figure S8).

To further explore the relationship between the peripheral (plasma/intestine) and central (brain) glutamatergic systems, the metabolic features of glutamate and its precursors (histidine, arginine, proline, and ornithine) were analyzed. L-glutamate and its important synthetic precursors (L-histidine and L-ornithine) were significantly increased in the hippocampus of FP chickens (*p* < 0.05; Figure S9). Glutamate levels were not statistically different in the amygdala, but DL-arginine levels were significantly increased (*p* < 0.05; Figure S10). Arginine (L-arginine and DL-arginine) and N-acetylornithine (a glutamate transformation product) levels were significantly increased in the plasma of FP chickens (*p* < 0.05) (Figure S11). In the cecum and duodenum, significant increases in glutamate metabolite (N-acetylglutamic acid and N-acetyl-DL-glutamic acid) and precursor (L-arginine) levels were observed, respectively (*p* < 0.05; Figures S12 and S13). There was no significant difference in the levels of substances related to glutamate synthesis and metabolism in the ileum of laying hens between the two groups (Figure S14). These results indicate the dysregulation of the glutamatergic system in FP chickens.

### 3.4. Transcriptome Profiling of Hippocampus and Amygdala Exhibits Distinct Gene Expression in FP Chickens

Transcriptome sequencing was used to explore gene expression differences affected by FP behaviors. Genes with a false discovery rate (FDR) ≤ 0.05 and fold change (FC) ≥ 1.5 were considered significantly differentially expressed. Using these criteria, 666 and 445 significantly differentially expressed genes were identified in the hippocampus and amygdala, respectively. Among them, 475 genes were significantly upregulated, and 636 genes were significantly downregulated (Table S6). Gene Ontology (GO) analysis was performed to understand the biological functions of these differentially expressed genes. GO analysis identifies associations between differentially expressed genes and specific cellular components, molecular functions, or biological processes. Fisher’s exact test with FDR correction for multiple testing was used. Figure S15 shows the top 30 enriched GO terms for significantly downregulated and upregulated genes in the hippocampus. These GO terms were mainly associated with biological processes. Downregulated genes were enriched in 22 biological processes, including the regulation of leukocyte-mediated immunity, the negative regulation of immune response, the sensory perception of sound, and the regulation of neurotransmitter levels. Upregulated genes were mainly enriched in biological processes such as feeding behavior, reproductive behavior, synaptic signaling, and the G protein-coupled receptor signaling pathway. As shown in Figure S16, downregulated amygdala genes were significantly enriched in GO, such as ion transport, regulation of natural killer cell-mediated immunity, and signaling receptor activity, while upregulated genes were mainly associated with behavior, the regulation of trans-synaptic signaling, and the G protein-coupled receptor signaling pathway.

**Figure 6:**
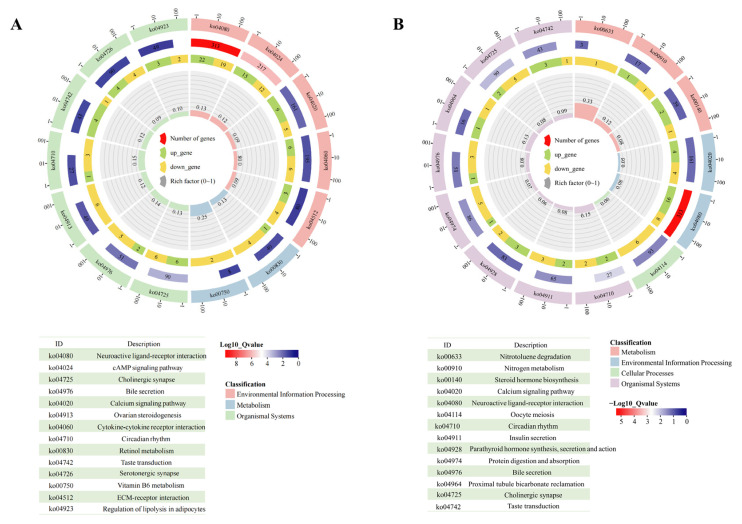
Pathway enrichment of differentially accumulated genes. KEGG cluster plot of differentially accumulated genes in the hippocampus (**A**) and amygdala (**B**). Each analysis involved six chickens. Permission has been obtained from Kanehisa Laboratories to use the KEGG pathway database [16].

KEGG pathway enrichment analysis was performed to infer gene functions mapped to biological pathways in the KEGG database. As shown in Figure 6A, hippocampal differential genes were significantly enriched in 12 KEGG pathways, including “Neuroactive ligand-receptor interaction, (ko04080)”, “cAMP signaling pathway (ko04024)”, “Cholinergic synapse (ko04725)”, and “Calcium signaling pathway (ko04020)”. To explore the expression of genes related to the glutamatergic system, the KEGG pathways “Arginine and proline metabolism (ko00330, *p* = 0.134)”, “histidine metabolism (ko00340, *p* = 0.252)”, “Glutamatergic synapse (ko04724, *p* = 0.093)”, and “GABAergic synapse (ko04727, *p* = 0.087)” were selected for further analysis. Four genes (*AGMAT*, *ALGH1A3*, *CARNS1*, and *P4HA3*) enriched in “arginine and proline metabolism” were significantly downregulated (Figure S17A). Two genes (*ALDH1A3* and *CARNS1*) enriched in “histidine metabolism” were also significantly downregulated (Figure S17A). Notably, genes enriched in the glutamatergic synapse, such as *GRIN2A* (encoding glutamate ionotropic receptor NMDA subunit 2A), *SLC17A6* (encoding vesicular glutamate transporter 2), and *KCNJ3* (encoding protein activates inwardly rectifying potassium channels 1), were significantly upregulated (*p* < 0.05). Amygdala differential genes were involved in nine KEGG pathways (Figure 6B), including “Neuroactive ligand-receptor interaction (ko04080)”, “Circadian rhythm (ko04710)”, “Cholinergic synapse (ko04725)”, and “Taste transduction (ko04742)”. Two genes related to the glutamatergic system, *ADCY1* (encoding adenylyl cyclase 1) and *GATM* (encoding glycine amidinyltransferase), were significantly downregulated (*p* < 0.05; Figure S17B).

### 3.5. Differential Gut Microbiomes Are Associated with Metabolites and Gene Expression Changes

The relationships between differential gut microbiota and metabolites were investigated using Spearman’s rank correlation analysis, with a selection criterion of |Rspearman| ≥ 0.6 and *p* < 0.05. As visualized in the correlation network (Figures S18–S20), there was a significant negative correlation between *Olsenella* and cortisol, N-acetylglutamate, and N-acetyl-DL-glutamate in the cecum. *Enterococcus* also exhibited a significant negative correlation with N-acetylglutamate and N-acetyl-DL-glutamate. In the duodenum, a significant negative correlation was observed between *Romboutsia* and L-arginine, N-acetyl-L-methionine, N-acetylvaline, L-epinephrine, and N-acetylalanine. In the ileum, a significant negative correlation was found between *Olsenella*, *Prevotellaceae_UCG_001*, *Prevotellaceae_Ga61_group*, and spermidine, 4-guanidinobutyric acid.

**Figure 7:**
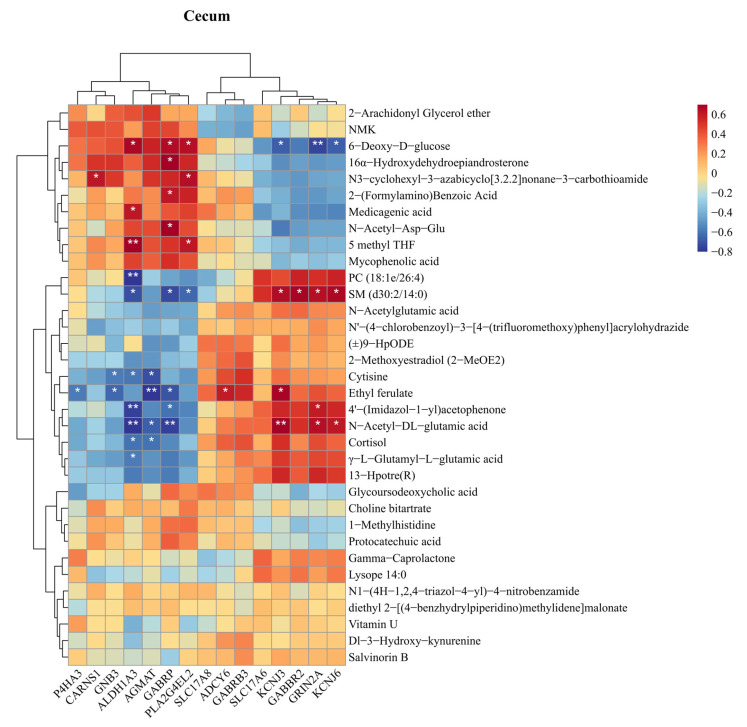
Correlation analysis of intestinal differential metabolites and hippocampal differential genes. Spearman correlation coefficients between the hippocampal differential genes and the differential metabolites in the cecum. * *p* < 0.05, ** *p* < 0.01.

To understand how gut-derived metabolites influence hippocampal gene expression, Spearman’s rank correlation was used to analyze the relationship between differential gut metabolites and glutamate-related genes. As shown in Figure 7, no significant correlation was observed between *SLC17A6* and differential cecal metabolites, but a significant positive correlation was observed between *GRIN2A* and N-acetyl-DL-glutamate (*p* < 0.05). In the duodenum (Figure S21), both *GRIN2A* and *SLC17A6* exhibited significant positive correlations with arginine and glycine-valine (*p* < 0.05). In the ileum (Figure S22), *SLC17A6* showed significant positive correlations with riboflavin and prostaglandin E1.

## 4. Discussion

### 4.1. The Hippocampal Glutamatergic System Affects FP in Laying Hens

In this work, we constructed an FP model in which the laying hens were subjected to chronic and unpredicted stress. Multi-omics analyses, including non-targeted metabolomics and eukaryotic transcriptome sequencing, demonstrated that the hippocampal glutamatergic system played a significant role in the development of FP. We observed that the levels of glutamate neurotransmitter and its precursors (i.e., histidine and ornithine) were significantly increased in the hippocampus of FP chickens. The expression of *GRIN2A* (encoding ionotropic glutamate receptor NMDA subunit 2A) and *SLC17A6* (encoding vesicular glutamate transporter 2) was also significantly upregulated in the hippocampus of FP chickens (Figure S17). Increasing evidence suggests that FP behavior is associated with depression and fear traits, and the glutamatergic system plays a vital regulatory role in the pathophysiology of depression and fear. The OF test results indicated that FP laying hens exhibited higher levels of depression and fear, manifested by a significant increase in latency to vocalize and ambulate and a reduction in walking steps (Figure 2).

Glutamate, an important excitatory neurotransmitter in the central nervous system, is widely distributed and highly concentrated in the hippocampus, cerebral cortex, and thalamus [17,18]. It plays a major role in shaping memory and learning and is closely associated with various neurological diseases such as depression and schizophrenia. Our present study found significantly high glutamate levels in the hippocampus of FP chickens, confirming our previous findings [9]. Most previous studies on FP have focused on the potential role of alteration in the central serotonergic and dopaminergic systems. They found that FP genotype and phenotype affected the serotonin metabolism of laying hens; low serotonin level was a possible reason for FP behavior of baby chicks, while high serotonin levels were observed in adult hens [7]. Dopamine metabolism in the central nervous system also differs significantly between low-FP and high-FP laying hens and is age-related [8,19]. In this study, serotonin (or dopamine) levels and related compounds did not differ significantly between pecking and neutral chickens, which may be related to the specific breeds used and indirectly indicates the complexity of the pecking mechanism.

Central neurons express various glutamate receptors, which are categorized into two main types: voltage-sensitive ionotropic glutamate receptors and ligand-sensitive metabotropic glutamate receptors. Glutamate receptors mediate excitatory synaptic transmission and are important for the maintenance of various functions such as learning and memory [20]. In this study, the expression of *GRIN2A* (NMDA receptor subunit 2A) and *SLC17A6* (encoding vesicular glutamate transporter 2) was significantly upregulated in the hippocampus of FP chickens. Previous studies also have observed significantly increased *GRIN2A* levels in the hippocampus of mice with depressive-like behavior [21]. Vesicular glutamate transporter 2 can activate the septohippocampal system, which is critical for learning adverse events and can lead to mood disorders such as aggression, aversion, and depression-related anhedonia [22].

In conclusion, the altered glutamatergic systems, such as the release of glutamate neurotransmitters and the upregulated expression of corresponding receptors, may be an important neuroregulatory mechanism of FP behavior in laying hens.

### 4.2. Gut Microbiota Regulates the Hippocampal Glutamatergic System Influencing FP Behavior in Laying Hens

The gut microbiota, sometimes referred to as the “second brain”, is intimately involved in various physiological processes such as host nutrient metabolism, and regulates the central nervous system, thus affecting host behaviors [23,24]. In the current study, we found that the composition of gut microbiota was significantly altered in FP chickens (Figure 4). Specifically, the relative abundance of *Sphingomonas* and *Pseudomonas* was significantly increased in the duodenum, and the abundance of bacterial genera such as *Romboutsia* and *Enterococcus* was significantly decreased (*p* < 0.05). The PICRUSt2-based functional prediction of bacterial community demonstrated that the gene expression of arginine and histidine biosynthesis was significantly increased (Figure S6). We found that the level of arginine in the FP chickens was significantly higher than that of neutral chickens, and there was a significant negative correlation between *Romboutsia* and arginine in the duodenum (Figure S19). High levels of *Romboutsia* have been observed in the feces of stress-resilient mice [25]. Arginine supplementation in the diet of birds significantly increased the relative abundance of ileal *Romboutsia*, indicating that the proliferation of gut *Romboutsia* may be determined by arginine metabolism [26]. Several studies have also shown that high or low dietary levels of arginine and methionine are associated with the occurrence of abnormal behaviors such as FP and anal pecking [27]. In the present study, although the histidine biosynthetic function was improved, the histidine level showed no obvious differences between the two groups of laying hens. This may be due to the fact that a large amount of histidine was consumed when they participated in the energy metabolism and antioxidant activity of intestinal epithelial cells exposed to environmental stressors [28].

Blood circulation is an important pathway for the gut microbiota to regulate the central nervous system through its metabolites. Our results indicated that the arginine level in the peripheral plasma was significantly increased; the histidine level also increased but not significantly (*p* = 0.076), which was probably due to the enhanced permeability of the BBB to histidine under environmental stressors [29]. Arginine and histidine supplementation increased their blood plasma concentrations [30,31]. Thus, in this study, the increased levels of plasma arginine and histidine can be mainly attributed to the enhancement of their biosynthesis in the gut bacteria, and the reduction in arginine metabolism. Previous studies have focused on the potential role of tryptophan and its metabolites and catecholamine hormones (e.g., dopamine, norepinephrine, and epinephrine) in FP behaviors of laying hens, while few studies have addressed the relationship between glutamate and its precursors and FP behaviors. Compared with gentle FP, the corticosterone level in severe FP laying hens was significantly decreased, while the epinephrine increased more significantly in the manual restraint test. A decreased level of tryptophan in peripheral blood, such as conversion into 5-HT [10], or the enhancement of tryptophan-KYN metabolic pathway [32], may be related to FP and aggressive behaviors in laying hens [33]. Similarly to previous results [34], we did not observe significant changes in the levels of tryptophan, 5-HT, and other metabolites in plasma, and analysis from ELISA revealed significantly increased levels of norepinephrine and epinephrine in plasma of FP chickens, indicating the stress state of FP chickens.

Metabolites in peripheral plasma can cross the BBB and ultimately affect animal behavior by regulating the central nervous system. Glutamate in the central nervous system is mainly derived from α-ketoglutaric acid in the citric acid cycle [17], and some studies have found that amino acids such as glutamine, arginine, proline, and histidine can also be converted to glutamate [12,35,36]. In addition, arginine and ornithine in the peripheral blood can cross the BBB into the central nervous system through the cationic amino acid transport protein 1 (CAT 1) [37,38], and histidine in the peripheral blood can cross the BBB through the large neutral amino acid transporter [39]. Our findings revealed a trend toward elevated levels of arginine and proline in the hippocampus; however, this difference did not reach statistical significance (*p* = 0.051 and 0.057, respectively). This is mainly attributed to the conversion of arginine and proline into glutamate [12], which also explains the significantly increased glutamate level observed in the hippocampus of FP chickens. Growing evidence indicates that the gut microbiota can regulate the central nervous system through the metabolism, and thus influence host behaviors. For example, the chronic treatment of mice with *Lactobacillus rhamnosus* could alleviate depressive and anxiety-like behaviors by producing gamma-aminobutyric acid (GABA) or altering the expression of GABA receptors [13]. In this study, the *GRIN2A* gene in the hippocampus exhibited a significant positive correlation with the levels of N-acetyl-glutamate in the cecum and arginine in the duodenum; there was a significant positive correlation between the *SLC17A6* gene and arginine in the duodenum and between riboflavin and prostaglandin E1 in the ileum (Figure 7, Figures S21 and S22). Elevated levels of arginine and riboflavin were also found in the peripheral blood of FP chickens, suggesting a potential role of these two differential metabolites. Arginine supplementation can activate central NMDA receptors through the arginine–nitric oxide pathway, causing depressive-like behavior in mice [40]. Riboflavin can inhibit the release of glutamate in the nervous system and affect glutamatergic neurotransmission, thereby exerting neuroprotective effects [41].

**Figure 8:**
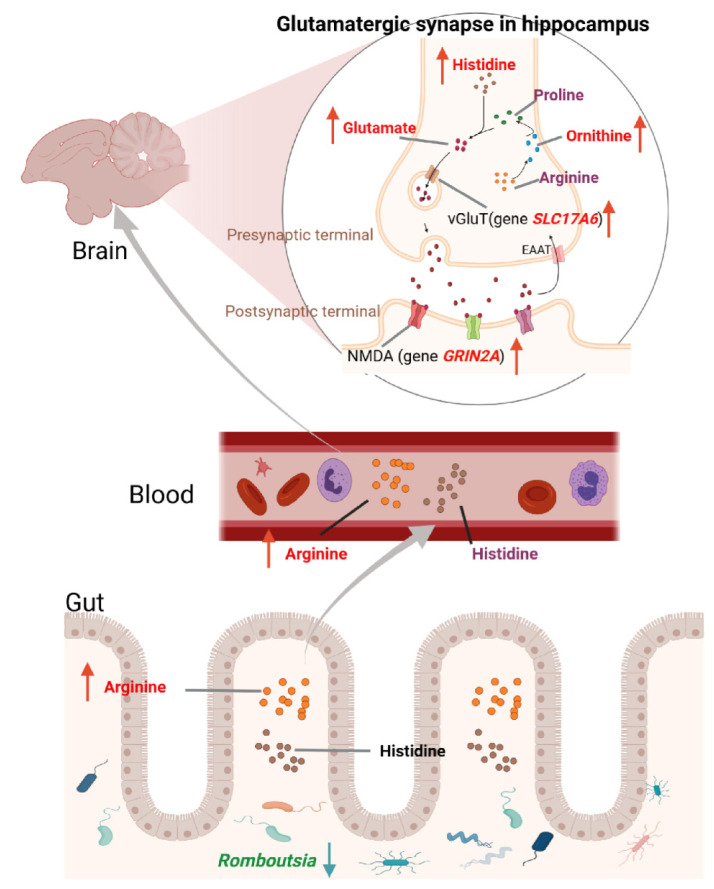
The proposed biological mechanism of FP behaviors. The gut microbiota regulates the glutamatergic system and affects FP in laying hens.

Therefore, we proposed a possible mechanism by which gut microbiota regulates the glutamatergic system to affect FP in laying hens, as shown in Figure 8. Gut microbiota such as *Romboutsia* may increase the levels of arginine and histidine in plasma by increasing the biosynthesis of arginine and histidine or reducing the metabolism of arginine, thereby increasing the levels of glutamate and expression of *GRIN2A* and *SLC17A6* genes in the hippocampus. The glutamatergic system was then altered in several brain regions associated with mood disorders such as anxiety and depression, eventually leading to FP behaviors. In this study, biological sampling was conducted only on the pecking chickens (feather peckers), following previous research. However, since pecked chickens (feather victims) are likely the most stressed, they should also be included in future analyses to provide a deeper understanding of the mechanisms behind FP.

## 5. Conclusions

This study revealed that FP in laying hens can reduce the production performance and cause potential immune system perturbation, concurrent with heightened fear responses and depressive-like behaviors. This may be related to alterations in gut microbiota composition and function, particularly changes in the abundance of *Romboutsia* and other genera in the intestine. These changes increase intestinal arginine and histidine biosynthesis and reduce arginine metabolism, leading to increased plasma arginine and histidine levels. Since both arginine and histidine can cross the BBB, they can be converted to glutamate in the hippocampus. This process increases *GRIN2A* and *SLC17A6* gene expression, thus regulating the central glutamatergic system and affecting FP behavior. While this study provides valuable insights into the correlations between differential gut microbiomes, metabolites, and gene expression changes, it is crucial to explore potential causal relationships to further understand the underlying mechanisms. Future investigations should be focused on the key microorganisms that affect FP in laying hens by regulating the central glutamatergic system through arginine and histidine. For instance, using germ-free animal models, fecal microbiota transplantation, and stable isotope tracing can provide deeper insights into how specific microbiome alterations influence metabolite production and gene expression over time. Overall, our findings provide theoretical support and new biological control strategies for reducing the occurrence of FP, especially severe FP, in the laying hen industry.

## Data Availability

Data used in this work are available via Zenodo (https://doi.org/10.5281/zenodo.10579290 (accessed on 29 January 2024)). This provides the original data for enzyme-linked immunosorbent assays, production performance, behaviors, transcriptomics, and metabolomics. In addition, 16s rRNA sequencing data that support the findings of this study have been deposited in the National Center for Biotechnology Information (https://www.ncbi.nlm.nih.gov/bioproject/PRJNA1069351 (accessed on 26 January 2024), accession: PRJNA1069351).

## Supplementary Materials

The following supporting information can be downloaded at: https://www.mdpi.com/article/10.3390/ani15091297/s1. Method S1: Analysis of non-targeted metabolomics data; Method S2: Analysis of 16S rRNA gene sequencing data; Method S3: Analysis of eukaryotic transcriptome sequencing data; Method S4: The open field test; Method S5: Production performance and egg quality; Method S6: Non-targeted metabolomic analysis; Table S1: The main instruments used in the experiments; Table S2: The main biochemical kits used in the experiments; Table S3: Types and descriptions of FP behaviors; Table S4: Quantification of immune response markers in two groups of laying hens; Table S5: The identified differential metabolite features in different biological organisms of feather peckers; Table S6: Differential genes in the hippocampus and amygdala of feather pecking chickens; Figure S1: Production performance of laying hens in two groups; Figure S2: Egg quality of laying hens in two groups; Figure S3: The relative abundance of gut microbiota at the phylum level; Figure S4: The relative abundance of gut microbiota at the genus level; Figure S5: The co-occurrence network of the core bacteria; Figure S6: Functional prediction of differential gut microbiota; Figure S7: The number of identified metabolic features in biological organisms; Figure S8: KEGG pathway enrichment analysis; Figure S9: The metabolic features of glutamate acid and its precursors in the hippocampus; Figure S10: The metabolic features of glutamate acid and its precursors in the amygdala; Figure S11: The metabolic features of glutamate acid and its precursors in the plasma; Figure S12: The metabolic features of glutamate acid and its precursors in the cecum; Figure S13: The metabolic features of glutamate acid and its precursors in the duodenum; Figure S14: The metabolic features of glutamate acid and its precursors in the ileum; Figure S15: The top 30 enriched GO terms for differential genes in the hippocampus of feather pecking chickens; Figure S16: The top 30 enriched GO terms for differential genes in the amygdala of feather pecking chickens; Figure S17: Analysis of differentially accumulated genes; Figure S18: The correlation network of cecum microbiota and metabolites; Figure S19: The correlation network of duodenum microbiota and metabolites; Figure S20: The correlation network of ileum microbiota and metabolites; Figure S21: Spearman correlation coefficients between the hippocampal differential genes and the differential metabolites in the duodenum; Figure S22: Spearman correlation coefficients between the hippocampal differential genes and the differential metabolites in the ileum.
