# Supplementary material for: The Glutamatergic System Regulates Feather Pecking Behaviors in Laying Hens Through the Gut–Brain Axis
Source: Animals (Basel). 2025 Apr 30;15(9):1297. doi: 10.3390/ani15091297 (PMC12071113; doi:10.3390/ani15091297)
Supplement: Supplementary file 1 [file animals-15-01297-s001.zip › animals-3599354-supplementary.pdf]

Supporting Information for

The Glutamatergic System Regulates Feather Pecking Behaviors in Laying Hens through the Gut–Brain Axis

Xiliang Yan[1], Chao Wang[1], Yaling Li[1], Yating Lin[1], Yinbao Wu[1], Yan Wang[1,2,3*]

[1] Heyuan Branch, Guangdong Laboratory for Lingnan Modern Agriculture, College of Animal Science, South China Agricultural University, Guangzhou 510642, China;

[2] Guangdong Provincial Key Lab of Agro-Animal Genomics and Molecular Breeding, South China Agricultural University, Guangzhou 510642, China;

[3] National Engineering Research Center for Breeding Swine Industry, College of Animal Science, South China Agricultural University, Guangzhou 510642, China

* Corresponding author:

Yan Wang, ywang@scau.edu.cn

Table of Contents

Method S1: Analysis of non-targeted metabolomics data··S4
Method S2: Analysis of 16S rRNA gene sequencing data···S4
Method S3: Analysis of eukaryotic transcriptome sequencing data·······························S5
Method S4: The open field test···S6
Method S5: Production performance and egg quality··S7
Method S6: Non-targeted metabolomic analysis··S7
Table S1: The main instruments used in the experiments··S10
Table S2: The main biochemical kits used in the experiments·····································S11
Table S3: Types and descriptions of FP behaviors··S12
Table S4: Quantification of immune response markers in two groups of laying hens···········S13
Table S5: The identified differential metabolite features in different biological organisms of feather peckers··S14
Table S6: Differential genes in the hippocampus and amygdala of feather pecking chickens······S15
Figure S1: Production performance of laying hens in two groups·····························S16
Figure S2: Egg quality of laying hens in two groups···S17
Figure S3: The relative abundance of gut microbiota at the phylum level·····················S18
Figure S4: The relative abundance of gut microbiota at the genus level························S19
Figure S5: The co-occurrence network of the core bacteria·····································S20
Figure S6: Functional prediction of differential gut microbiota································S21
Figure S7: The number of identified metabolic features in biological organisms··············S22
Figure S8: KEGG pathway enrichment analysis···S23
Figure S9: The metabolic features of glutamate acid and its precursors in the hippocampus·······S24
Figure S10: The metabolic features of glutamate acid and its precursors in the amygdala·····S25
Figure S11: The metabolic features of glutamate acid and its precursors in the plasma······S26
Figure S12: The metabolic features of glutamate acid and its precursors in the cecum········S27
Figure S13: The metabolic features of glutamate acid and its precursors in the duodenum····S28
Figure S14: The metabolic features of glutamate acid and its precursors in the ileum·········S29
Figure S15: The top 30 enriched GO terms for differential genes in the hippocampus of feather pecking chickens··S30
Figure S16: The top 30 enriched GO terms for differential genes in the amygdala of feather pecking chickens··S32
Figure S17: Analysis of differentially accumulated genes··S33

Figure S18: The correlation network of cecum microbiota and metabolites····················S34
Figure S19: The correlation network of duodenum microbiota and metabolites················S35
Figure S20: The correlation network of ileum microbiota and metabolites······················S36
Figure S21: Spearman correlation coefficients between the hippocampal differential genes and the differential metabolites in the duodenum···S37
Figure S22: Spearman correlation coefficients between the hippocampal differential genes and the differential metabolites in the ileum···S38

Method S1. Analysis of Non-targeted Metabolomics Data.

The KEGG, HMDB, and LIPIDMaps databases were used as reference resources for identified metabolites annotation. For the multivariate statistical analysis, the metaX software was first used to preprocess the metabolomics data. Then, principal component analysis (PCA) and partial least squares discriminant analysis (PLS-DA) were performed to obtain the VIP value of each metabolite. For the univariate analysis, a *t*-test was used to calculate the statistical significance (P value) of each metabolite between the two groups, and the fold change (FC value) was also analyzed. The default criteria for differential metabolite screening were VIP > 1 and $P < 0.05$. The ggplot2 package in R was used to create a bubble chart, and the metabolite function and pathway were analyzed by MetaboAnalyst 5.0 (https://www.metaboanalyst.ca/). The metabolite pathway was considered to be enriched if x/n > y/n.

Method S2. Analysis of 16S rRNA Gene Sequencing Data.

In accordance with barcode sequence and PCR amplification primer sequence, the sample data were split from the offline data. After truncating the barcode and primer sequences, the FLASH software (V1.2.11) was used to splice the sample reads to obtain raw tags. The fastp software was then used to perform quality control on the obtained raw tags to obtain high-quality clean tags. The Vsearch software was used to compare clean tags with the database to detect and remove chimeras and to obtain effective tags.

The DADA2 module in QLIME2 software was used to obtain the amplicon sequence variants (ASVs) and the feature table by filtering out sequences with an abundance of less than 5. Subsequently, the resulting ASVs were aligned to the database using the classify-sklearn module in the QIIME2 software to obtain the species information. According to the resulting feature table and ASV species annotation information, the statistical analysis was performed on two groups of datasets.

The QIIME2 software was used to calculate observed_otus, shannon, simpson, chao1, woods_coverage, dominance, and pielou_e indices and to draw the rarefaction curve and species accumulation boxplots. The LEfSe software was used to perform significant differential species analysis.

Method S3. Analysis of Eukaryotic Transcriptome Sequencing Data.

(1) Gene expression analysis

Gene expression levels are generally measured by how much mRNA is transcribed. First, the feature count software (http://subread.sourceforge.net/) was used to analyze the read information of each gene. Here, two methods (FPKM and TPM), as described below, were used for normalization analysis.

FPMK = $\frac{total\ exon\ Fragments}{mapped\ reads\ (Millons) \times exon\ length\ (KB)}$

TPM = $\frac{\frac{N_i}{L_i} * 10^6}{sum\ (\frac{N_1}{L1} + \frac{N_2}{L2} + \cdots + \frac{N_n}{Ln})}$

where N_i is the number of reads aligned to the *i*th exon and L_i is the length of the *i*th exon.

(2) Differential analysis of gene expression

The edgeR package in R3.6.3 was used to calculate the statistical significance of each gene expression between the two groups. The criteria of the differential gene were set as FC ≥ 1.5 and FDR ≤ 0.05.

(3) Differential gene annotation and enrichment analysis

The KEGG database was used for differential gene annotation. The KOBAS and Goatools software were, respectively, used to perform KEGG pathway enrichment analysis and GO enrichment analysis. Fisher's exact test was used to examine the significance of the association. In order to control the calculated false positive rate, multiple tests were performed using the BH (FDR) method, with a P value of 0.05 as the threshold. KEGG pathways or GO pathways that meet this condition are defined as significantly enriched in differentially expressed genes.

Method S4. The Open Field Test.

As an experimental measure of exploratory behavior and general activity in animals, the open field (OF) test was used to evaluate the depression (or fear)-related activity of chickens at 31 weeks of age. For the OF test, each chicken was first placed at the center of a wooden fence (120 cm × 120 cm × 150 cm) and then tested for 5 minutes. A camera above the OF was used to record the chicken behavior, including latency to vocalize, the number of vocalizations, latency to ambulate, and the number of steps. All OF tests were performed over

two consecutive nights (20:00 to 24:00) and were recorded by three trained observers.

Method S5. Production Performance and Egg Quality.

The production performance of laying hens was determined by the following indicators:

(1) Average daily feed intake (g) = total feed consumption/experimental days;

(2) Egg laying rate (100%) = number of eggs produced/chickens per cage × 100%;

(3) Total egg weight = total egg weight produced by chickens in each cage during the experimental period;

(4) Average egg weight = total egg weight/number of eggs produced;

(5) Feed conversion ratio = total feed consumption/total egg weight.

At the same time, we collected eggs from laying hens during the period of 30 to 32 weeks and analyzed the egg quality characteristics as follows:

(6) Egg shape index = short diameter of the egg/the long diameter of the egg;

(7) Haugh unit = $100\times\log10(H-1.7\times W^{0.37}+7.57)$, where H is observed height of the albumen (mm) and W is the egg weight (g);

(8) Eggshell thickness (μm) = the average thickness of the blunt end, equator and pointed end of the eggshell;

(9) Eggshell strength (kg/cm^2): measured by the Egg Shell Strength Tester.

Method S6. Non-targeted Metabolomic Analysis.

Intestinal sample preparation: Firstly, 100 mg of intestinal sample was accurately weighed and filled into a 2 mL centrifuge tube. Then, 600 μL of methanol (containing 2-chloro-L-phenylalanine) was added and vortexed for 30 s. After vortex mixing, the sample was ground in a tissue grinder for 90 s at 60 Hz and ultrasonicated for 10 min at room temperature. Next, the sample was centrifuged for 10 min at 12,000 rpm and 4°C. Afterward, the supernatant was filtered through a 0.22 μm membrane, and the filtrate was transferred to the headspace vials for LC-MS (liquid chromatography–mass spectrometry) analysis.

Plasma sample preparation: Plasma samples were thawed at 4°C and subsequently vortexed for 1 min. Then, 20 μL of plasma sample was accurately taken into a 2 mL centrifuge tube, followed by vortexing with 400 μL methanol for 1 min. After vortexing, the solution was centrifuged for 10 min at 12,000 rpm and 4°C. The supernatant was transferred to a 2 mL centrifuge tube and concentrated to dryness. Then, the sample was reconstituted in 150 μL of 2-chloro-L-phenylalanine (4 ppm), and the supernatant was filtered through a 0.22 μm membrane. The filtrate was finally transferred to the headspace vials for LC-MS analysis.

Brain tissue sample preparation: At first, about 50 mg of brain tissues were accurately weighed into a 2 mL centrifuge tube and 1000 μL of tissue extraction reagent (75% 9:1 methanol/chloroform, 25% H_2O) was accurately added. Then, the sample was ground in a tissue grinder for 60 s at 50 Hz, and ultrasonicated

for 30 min at room temperature. Next, the sample was centrifuged for 10 min at 12,000 rpm and 4°C. The supernatant was transferred to a 2 mL centrifuge tube and concentrated to dryness. The sample was reconstituted in 200 μL of 2-chloro-L-phenylalanine (4 ppm), after which the supernatant was filtered through a 0.22 μm membrane. Finally, the filtrate was transferred to the headspace vials for LC-MS analysis.

The prepared samples were analyzed on an Ultra Performance Liquid Chromatography system (Vanquish, Thermo Fisher Scientific, USA) with an ACQUITYUPLC®HSST3 column (2.1×150 mm, 1.8 μm) coupled to a ThermoQ Exactive mass spectrometer. In positive ionization mode, the mobile phase consisted of 0.1% formic acid acetonitrile (C) and 0.1% formic acid in water (D). Elution gradient was set as 0~1 min, 2% C; 1~9 min, 2%~50% C; 9~12 min, 50%~98% C; 12~13.5 min, 98% C; 13.5~14 min, 98%~2% C; and 14~20 min, 2% C. In negative ionization mode, the mobile phase consisted of acetonitrile (A) and 5 mM ammonium formate in water (B). Elution gradient was set as 0~1 min, 2% A; 1~9 min, 2%~50% A; 9~12 min, 50%~98% A; 12~13.5 min, 98% A; 13.5~14 min, 98%~2% A; and 14~20 min, 2% A. Mobile phase flow rate: 0.25 mL/min; column temperature: 40℃; and injection volume: 2 μL. The electrospray ionization (ESI) source parameters were set as follows: sheath gas flow rate, 30 arb; aux gas flow rate, 10 arb; capillary temperature, 325 ℃; and full MS resolution, 70000. Non-targeted metabolomic analysis was performed by Beijing Novogene Biotechnology Co., Ltd (Beijing, China).

Table S1. The main instruments used in the experiments.

Equipment Name	Product Model	Manufacturer
Video Camera	DS-7080N-K2	Hangzhou Hikvision Digital Technology Co., Ltd. (China)
Network Camera	DS-2CD2T55(D)-I3	Hangzhou Hikvision Digital Technology Co., Ltd. (China)
PCR Amplifier	C1000TM Thermal Cycler	Thermo (America)
Electrophoresis apparatus	DYY-6D	Beijing Liuyi Biotechnology Co., Ltd. (China)
Gel Imager	Bio-Best 200E	SIM (America)
Nucleic acid concentration measurement system	QubitTM3 Fluorometer	Thermo (MA, America)
Low-temperature High-speed Centrifuge	D-37520	Thermo (MA, America)
Grinding Machine	JXFSTPRP-48	Shanghai Jingxin Industrial Development Co., Ltd. (China)
Full wavelength microplate reader	Mutiskan go	Thermo (MA, America)
Eggshell Strength Tester	ESG-1	Nanjing Yaoen Instruments and equipment Co., Ltd. (China)
Egg Albumen Height Tester	YN-11L	Nanjing Yaoen Instruments and equipment Co., Ltd. (China)
Sequencing Systems	Illumina NovaSeq	Illumina (CA, America)
Liquid Chromatograph	Vanquish	Illumina (CA, America)
Mass spectrometer	Q Exactive	Illumina (CA, America)

Table S2. The main biochemical kits used in the experiments.

Product Name	Manufacturer
ELISA Kit	Shanghai Enzyme linked Biotechnology Co., Ltd (Shanghai, China)
QIAamp PowerFecal DNA Kit	Qiagen (Hilden, Germany)
RNAprep pure Tissue Kit	Tiangen Biotech (Beijing) Co., Ltd. (China)
2×Taq PCR Mix (KT201-02)	Tiangen Biotech (Beijing) Co., Ltd. (China)
PCR primers	Sangon Biotech (Shanghai) Co., Ltd. (China)

Table S3. Types and descriptions of FP behaviors.

Types	Descriptions
Severe FP	One chicken grabs, pulls, or tears another chicken's feathers with its beak, causing the feathers to break or be pulled out.
Exploratory FP	One chicken slightly touches another chicken's feathers with its beak, but not significantly changing the position of the feathers.
Stereotypical FP	A round of stereotypical FP is defined as three or more slight pecks on a single body part within one second.

Table S4. The plasma concentrations of immune response markers in two groups of laying hens. Each group consisted of six chickens.

Biomarkers	Feather peckers	Neutrals	*P* value	Sampling time
IL-1 (pg/mL)	231 ± 6.21	289 ± 6.61	0.000	Four weeks after the imposition of multiple stressors
IL-6 (pg/mL)	26.2 ± 1.64	27.9 ± 0.630	0.040	
TNF-α (pg/mL)	57.1 ± 5.53	67.0 ± 3.91	0.005	
IgA (ng/mL)	258 ± 14.4	274 ± 11.0	0.050	
IgG (ng/mL)	2175 ± 86.8	2540 ± 266	0.018	
IgM (ng/mL)	574 ± 50.8	630 ± 22.9	0.033	
CORT (pg/mL)	110 ± 5.72	103 ± 19.5	0.408	
EPI (pg/mL)	658 ± 27.1	551 ± 37.1	0.000	
NE (pg/mL)	1316 ± 54.3	1103 ± 74.2	0.000	

Table S5. The identified differential metabolite features in different biological organisms of feather peckers. Each analysis involved six chickens.

Organisms	Upregulated metabolites	Downregulated metabolites	Total metabolites
hippocampus	82	39	121
amygdala	19	9	28
plasma	32	49	81
cecum	18	16	34
duodenum	24	17	41
ileum	60	12	72

Table S6. Differential genes in the hippocampus and amygdala of feather pecking chickens. Each analysis involved six chickens.

Organisms	Upregulated genes	Downregulated genes	Differential genes	Total
hippocampus	232	434	666	16643
amygdala	243	202	445	16597

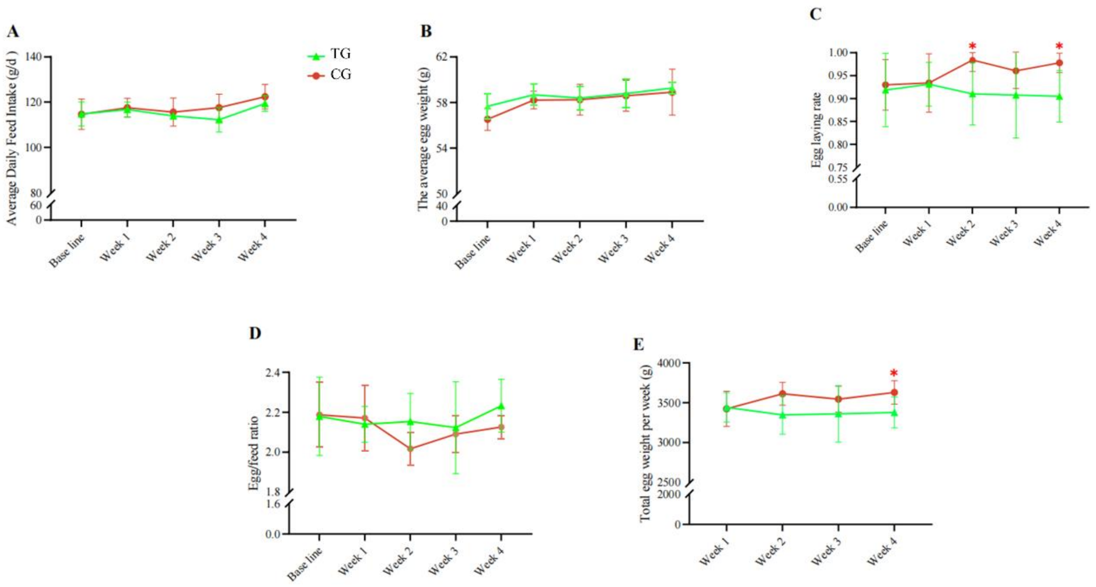

Figure S1. Production performance of laying hens in two groups. TG, treatment group; CG, control group. * $P < 0.05$, ** $P < 0.01$. Each group consisted of six chickens.

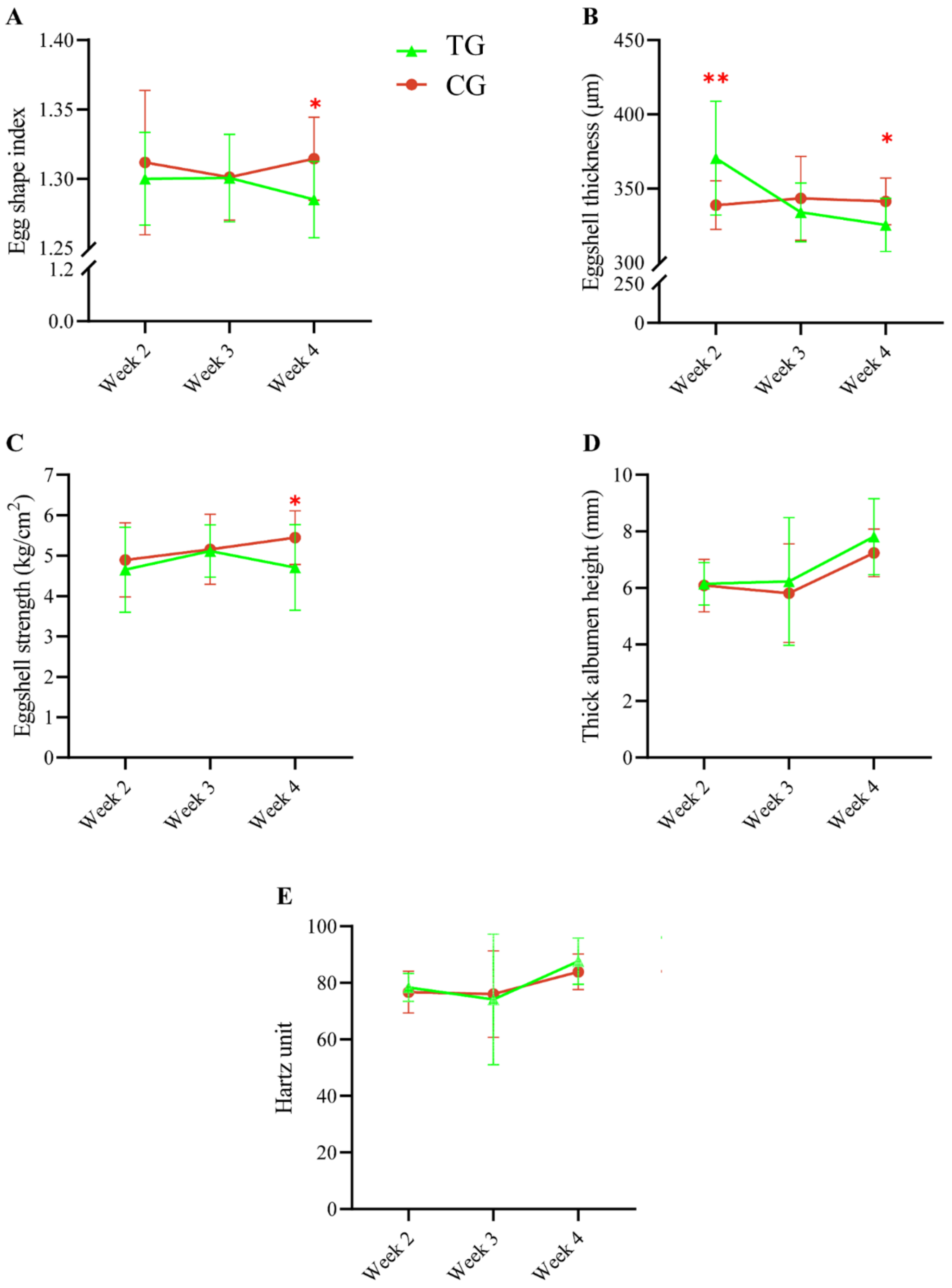

Figure S2. Egg quality of laying hens in two groups. TG, treatment group; CG, control group. * $P < 0.05$, ** $P < 0.01$. Each group consisted of six chickens.

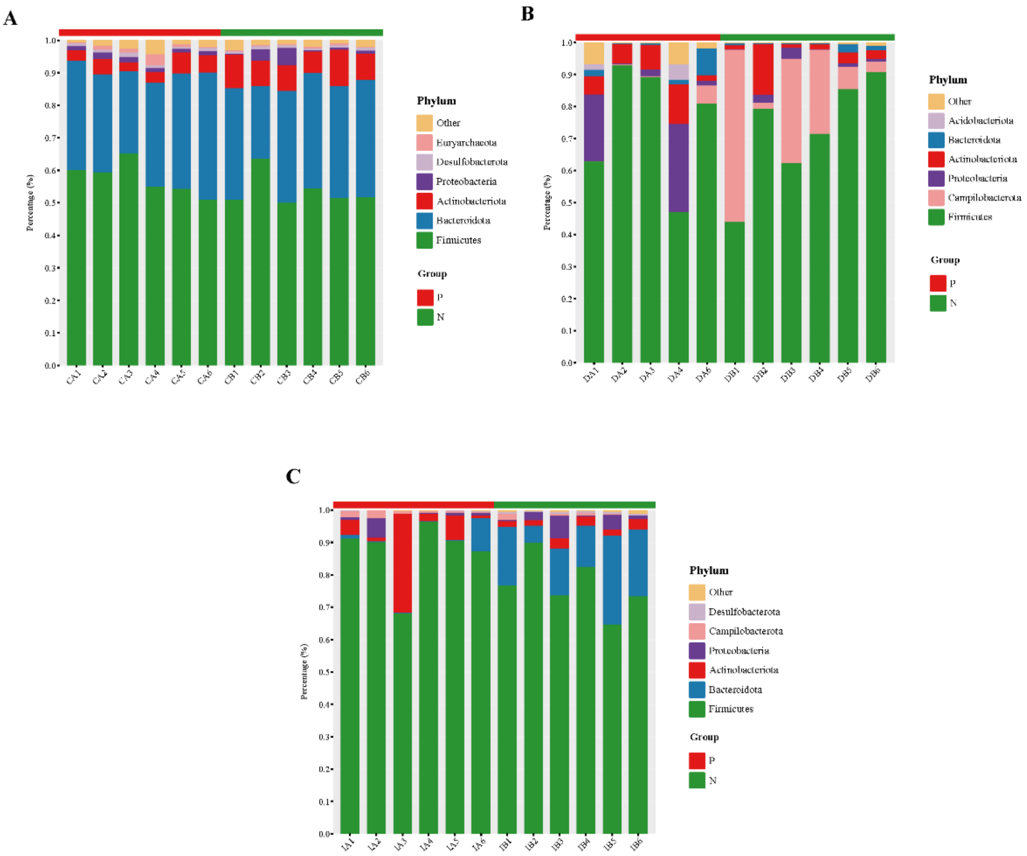

Figure S3. The relative abundance of gut microbiota at the phylum level. Cecum (**A**), duodenum (**B**), ileum (**C**). P, feather peckers; N, neutral chickens. Each group consisted of six chickens.

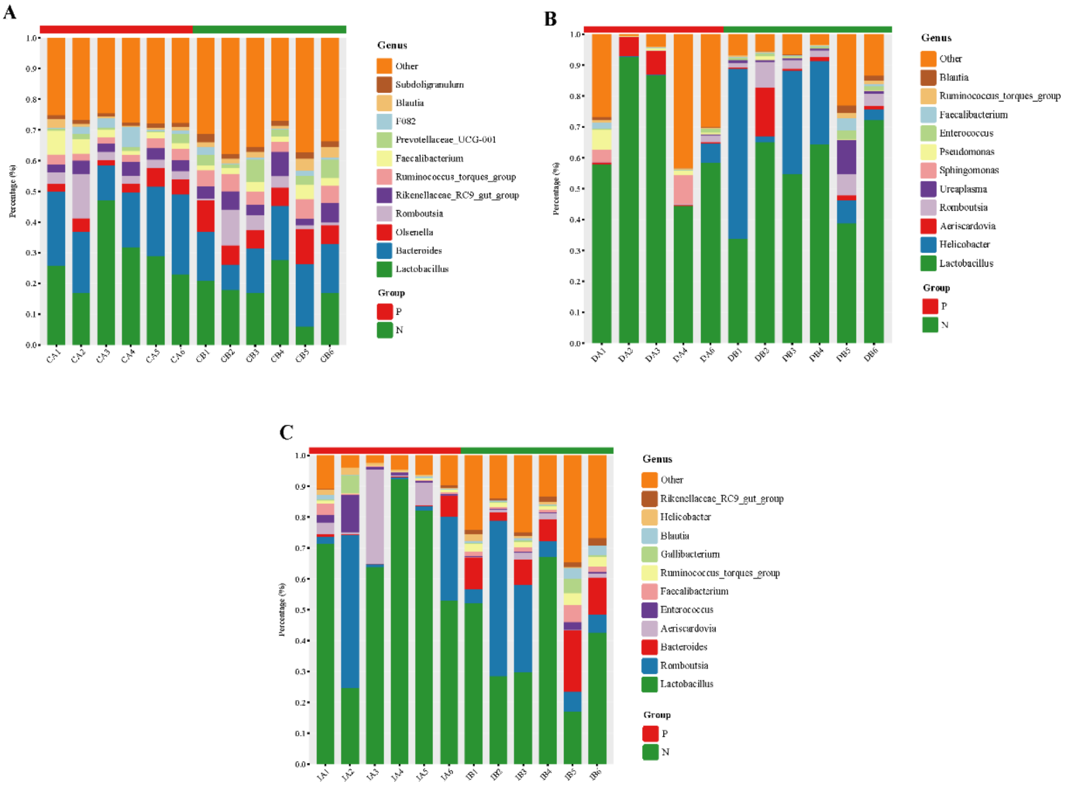

Figure S4. The relative abundance of gut microbiota at the genus level. Cecum (**A**), duodenum (**B**), ileum (**C**). P, feather peckers; N, neutral chickens. Each group consisted of six chickens.

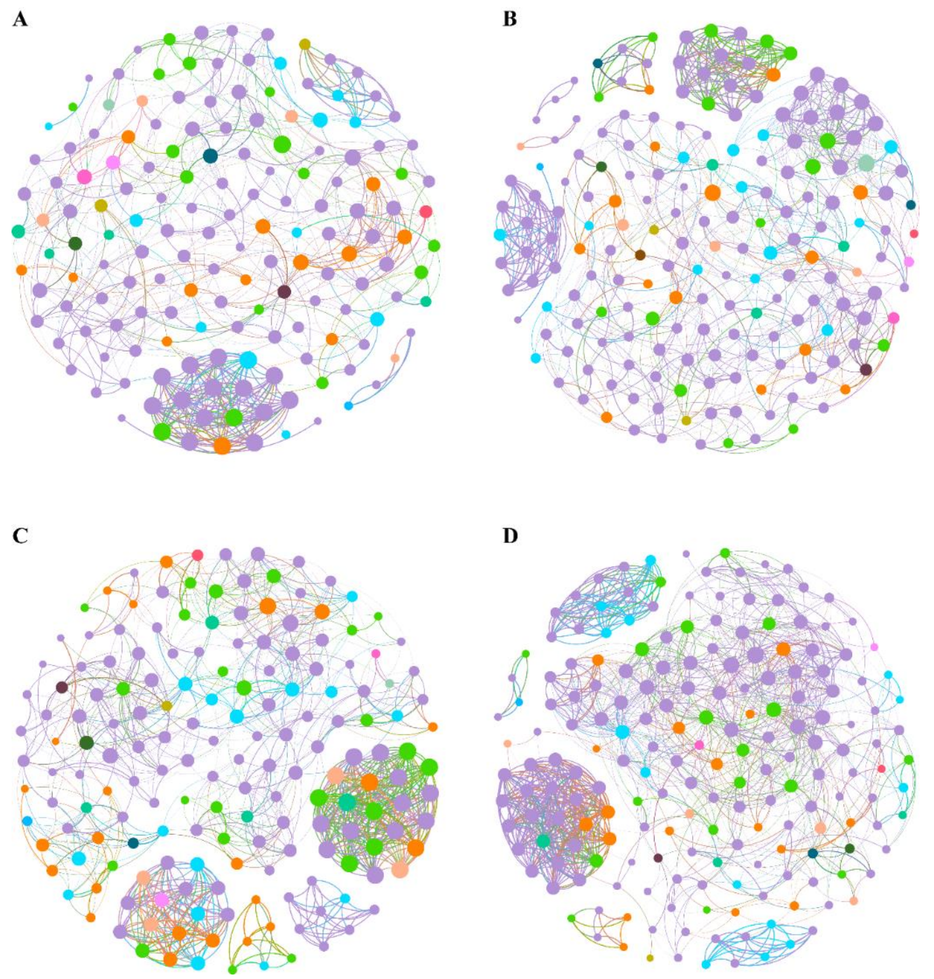

Figure S5. The co-occurrence network of the core bacteria in the cecum of feather peckers (**A**) and neutral chickens (**B**), and the ileum of feather peckers (**C**) and neutral chickens (**D**). Gut microbiota at the same phylum level was labeled as the same color. Each group consisted of six chickens.

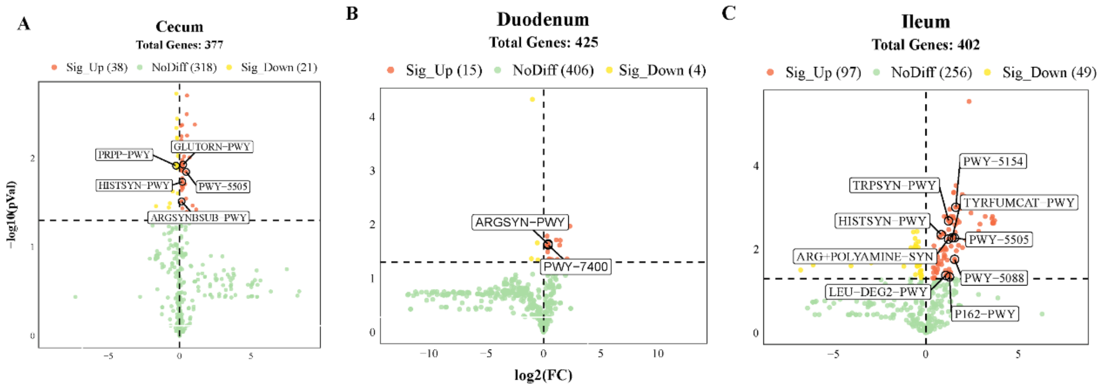

Figure S6. Functional prediction of differential gut microbiota. Volcano plot of bacteria communities at the genus level in the cecum (**A**), duodenum (**B**), and ileum (**C**). In the volcano plots, orange dots represent upregulated expressed genes, yellow dots represent upregulated expressed genes, and green dots represent genes that are not differentially expressed. Each analysis involved six chickens.

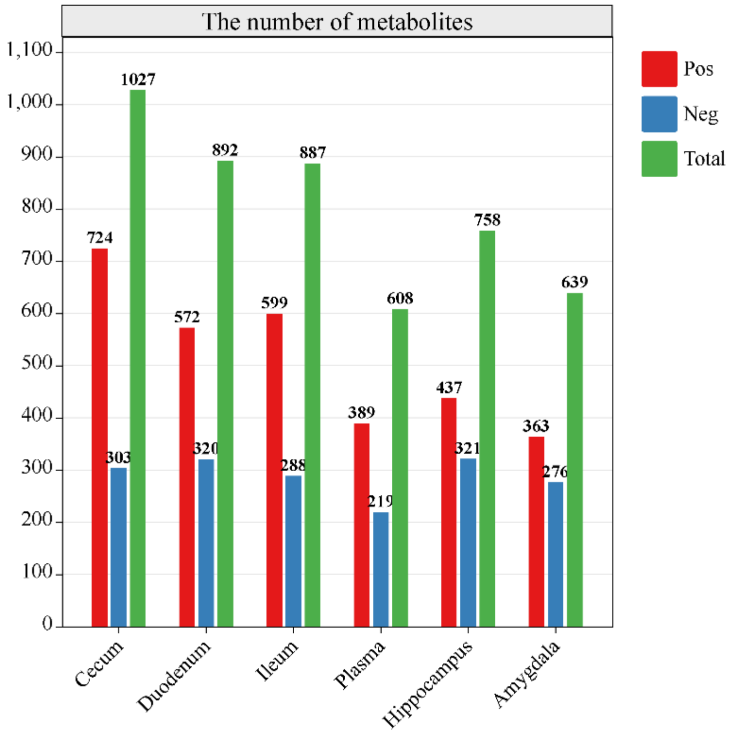

Figure S7. The number of identified metabolic features in biological organisms. Pos, positive ion mode; Neg, negative ion mode. Each analysis involved six chickens.

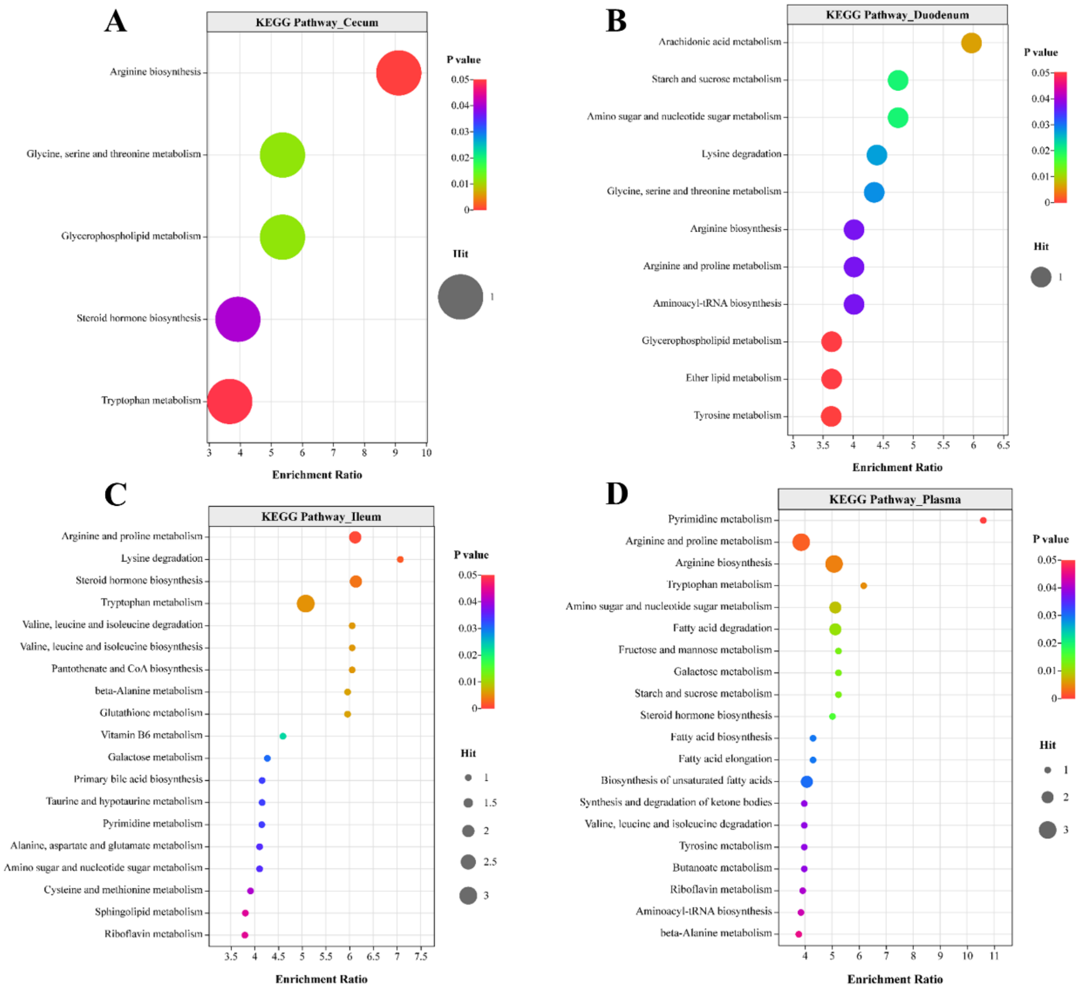

Figure S8. KEGG pathway enrichment analysis. Pathway enrichment of differentially accumulated metabolites in the cecum (**A**), duodenum (**B**), ileum (**C**), and plasma (**D**). The bubble size represents the number of metabolites; the color bar represents the corrected *P* value. Each analysis involved six chickens. Permission was obtained from Kanehisa Laboratories to use the KEGG pathway database[1].

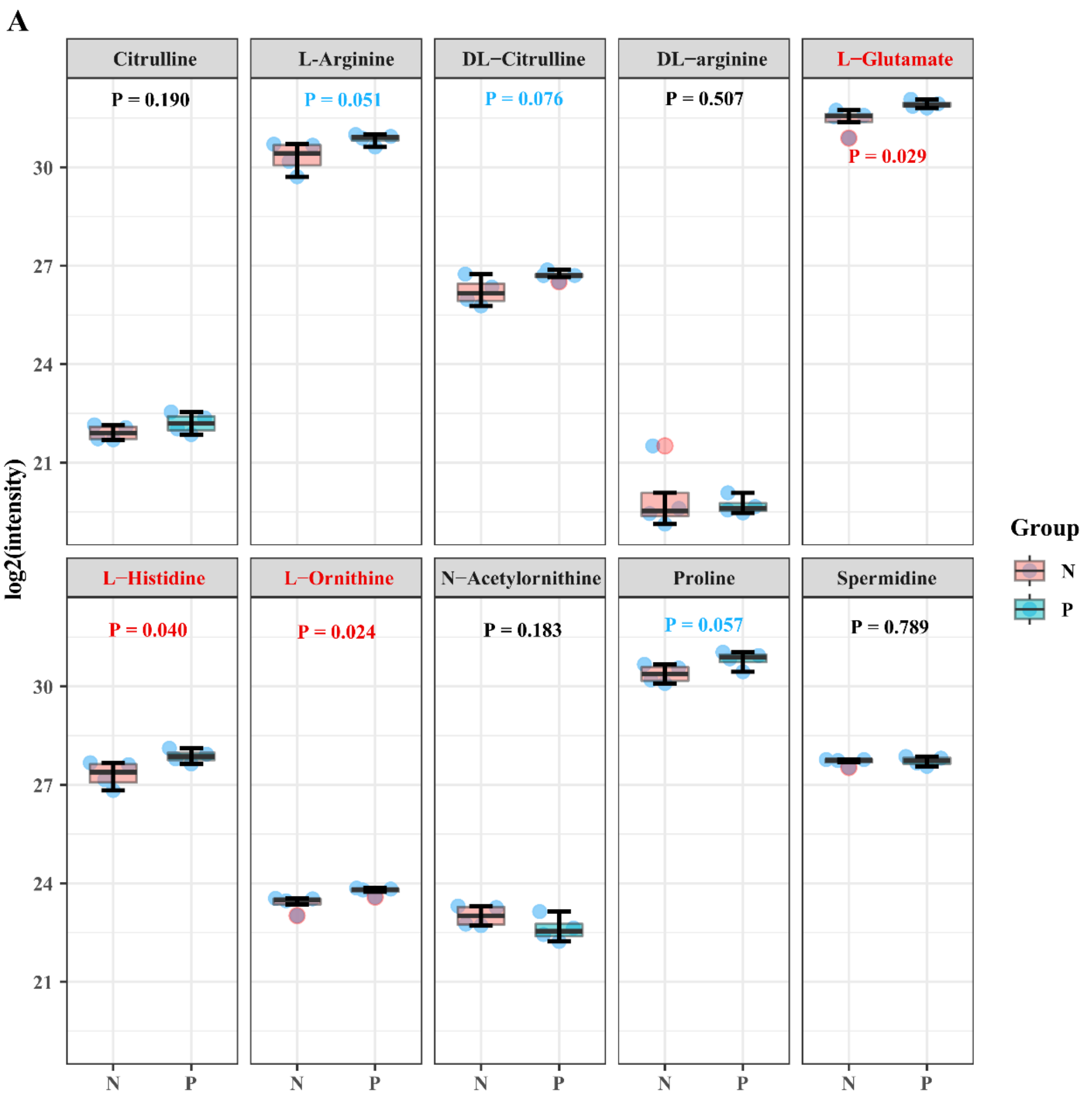

Figure S9. The metabolic features of glutamate acid and its precursors in the hippocampus. P, feather peckers; N, neutral chickens. Each group consisted of six chickens.

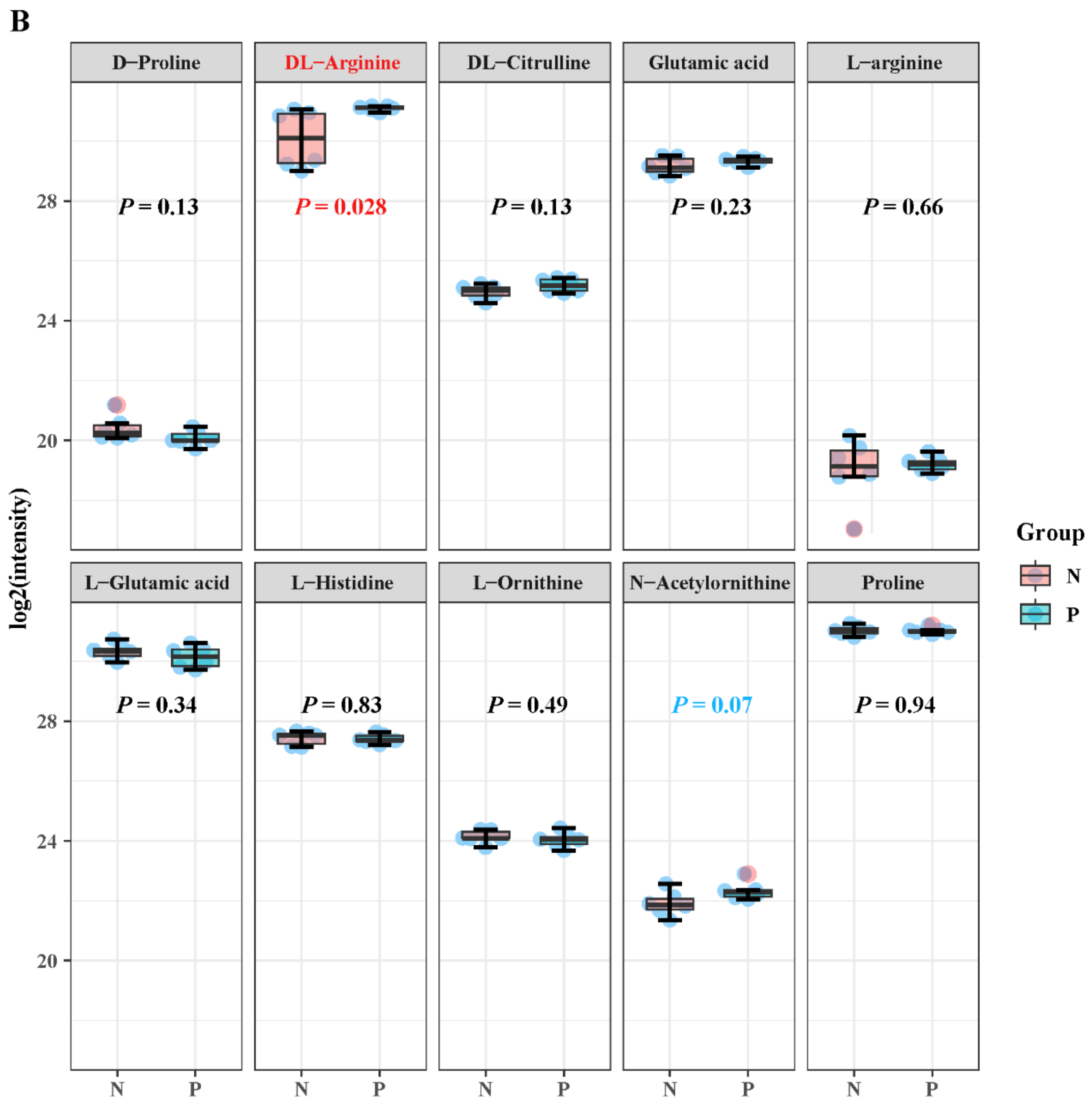

Figure S10. The metabolic features of glutamate acid and its precursors in the amygdala. P, feather peckers; N, neutral chickens. Each group consisted of six chickens.

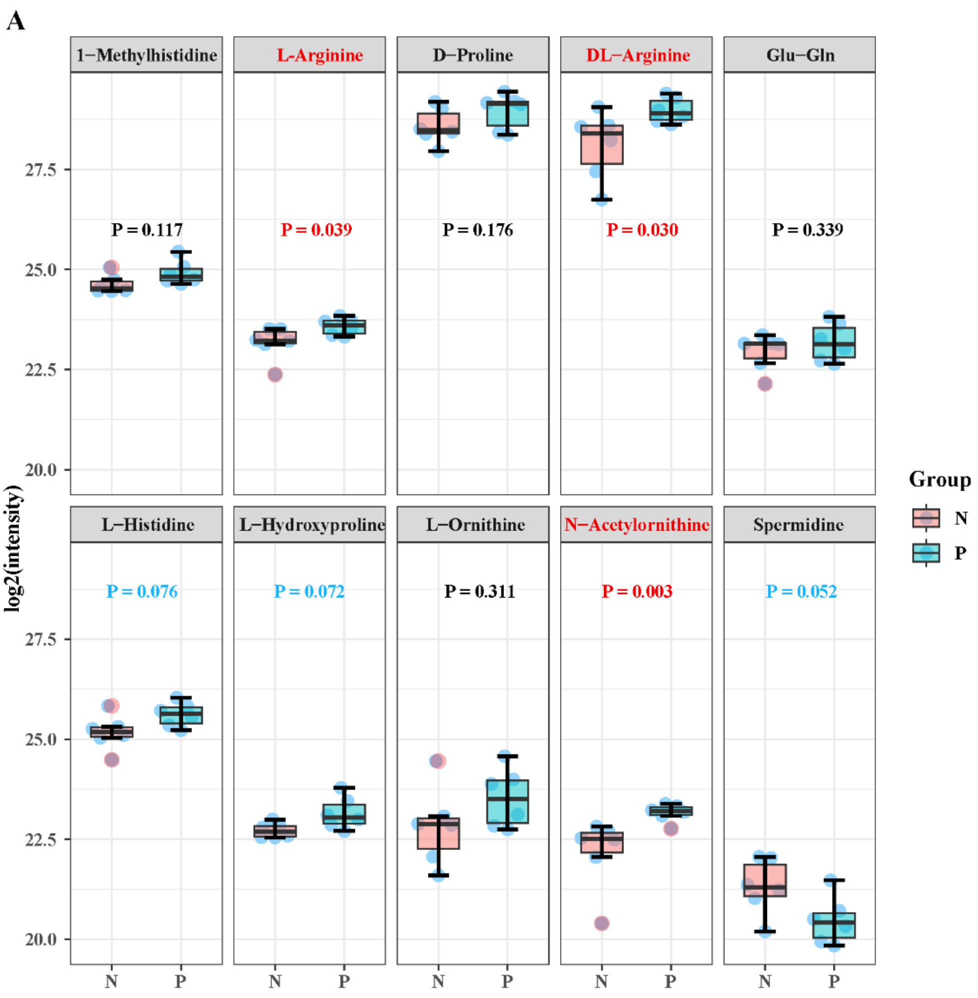

Figure S11. The metabolic features of glutamate acid and its precursors in the plasma. P, feather peckers; N, neutral chickens. Each group consisted of six chickens.

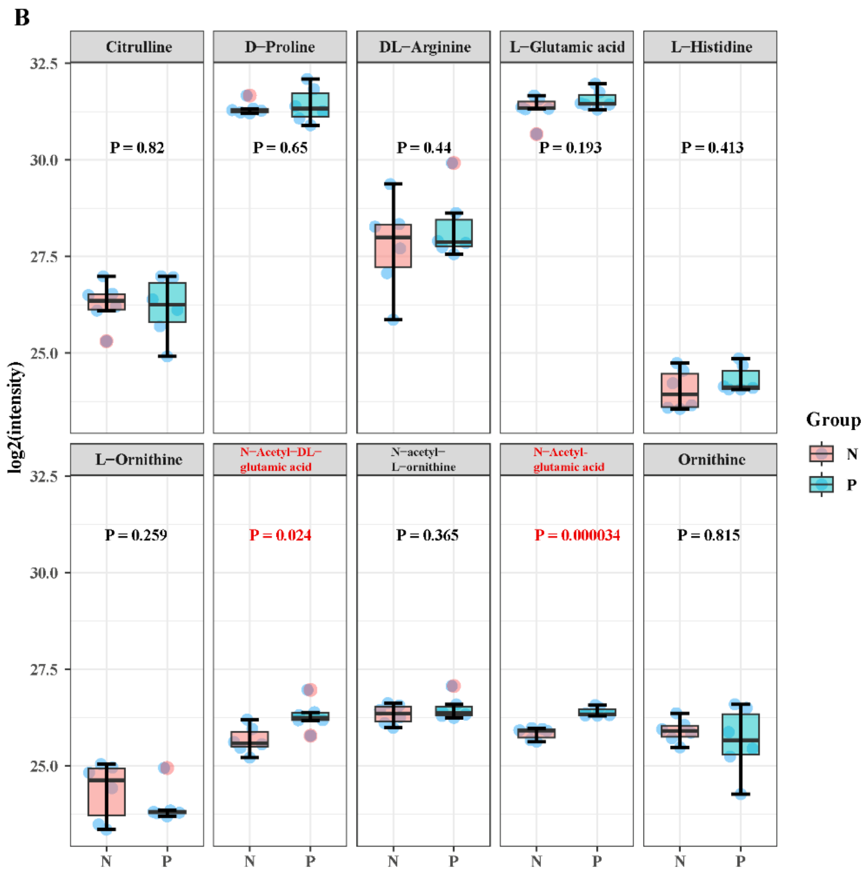

Figure S12. The metabolic features of glutamate acid and its precursors in the cecum. P, feather peckers; N, neutral chickens. Each group consisted of six chickens.

C

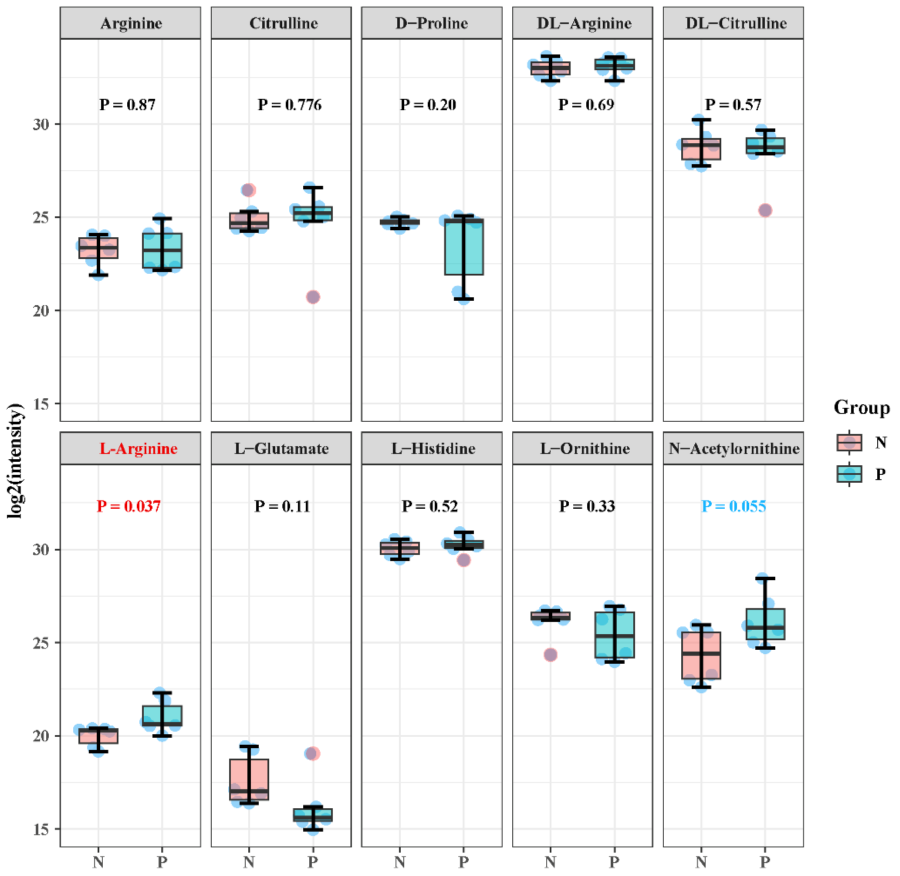

Figure S13. The metabolic features of glutamate acid and its precursors in the duodenum. P, feather peckers; N, neutral chickens. Each group consisted of six chickens.

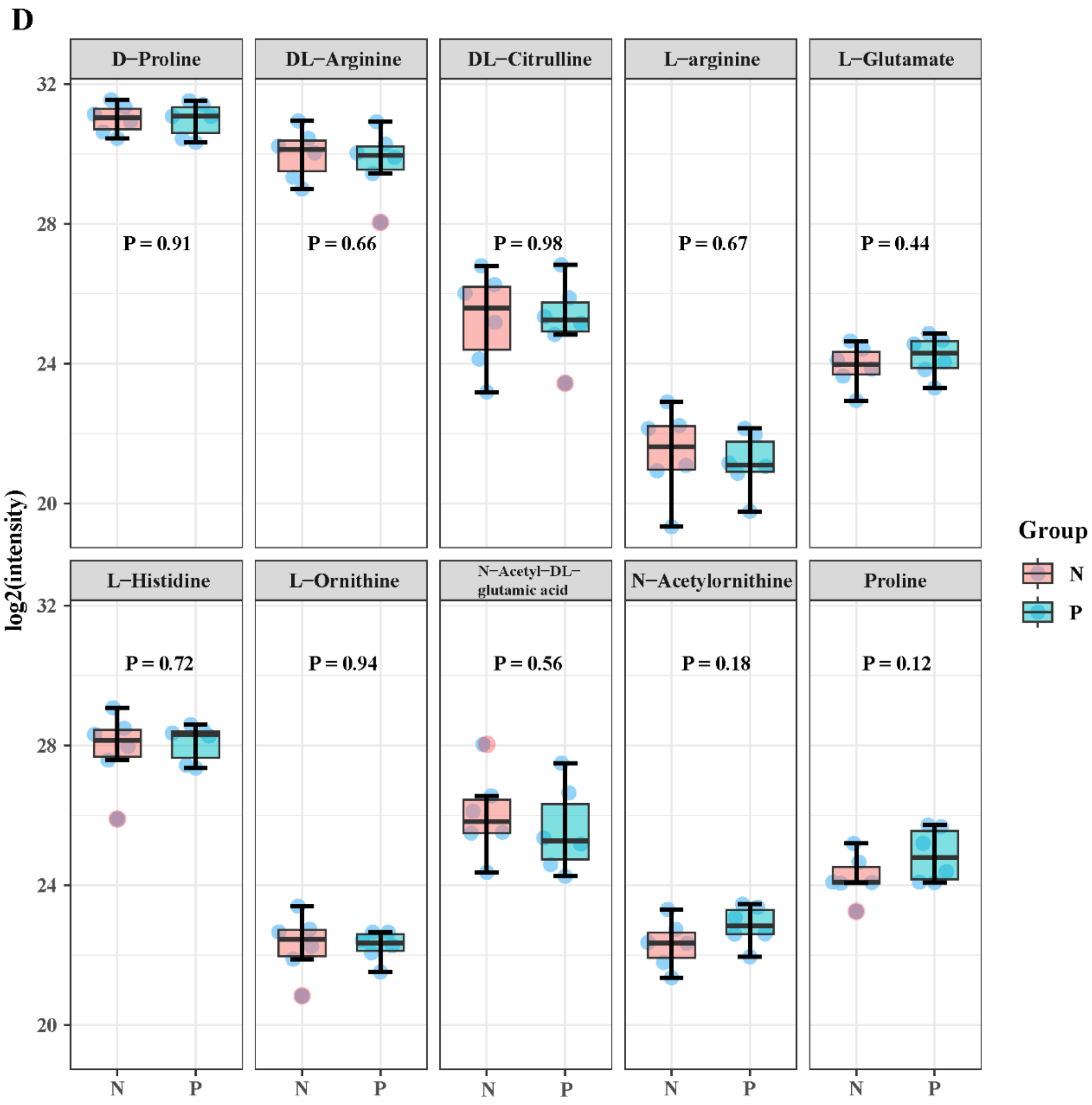

Figure S14. The metabolic features of glutamate acid and its precursors in the ileum. P, feather peckers; N, neutral chickens. Each group consisted of six chickens.

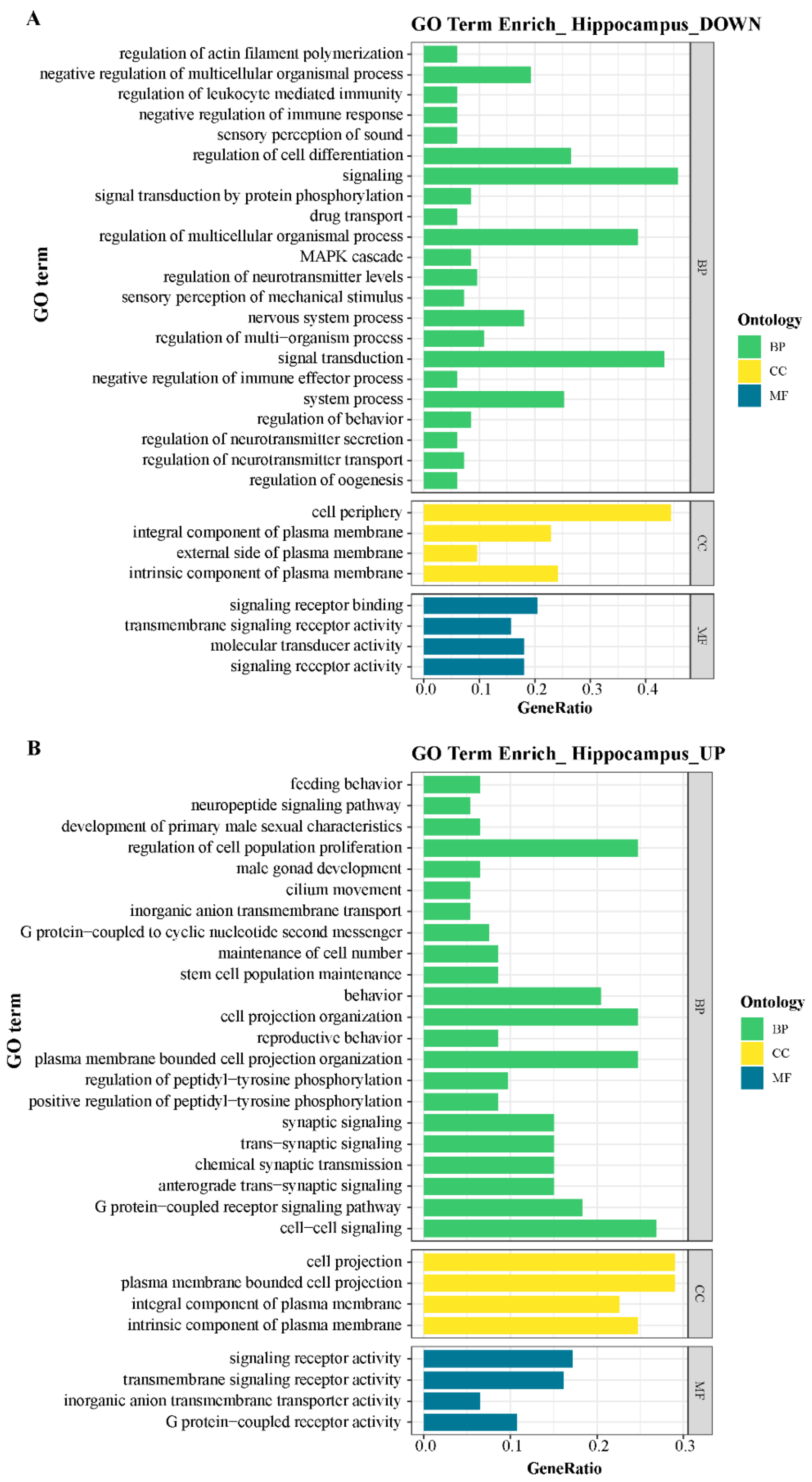

Figure S15. The top 30 enriched GO terms for significantly downregulated (**A**) and upregulated (**B**) genes in the hippocampi of feather pecking chickens. BP, biological process; CC, cellular component; MF, molecular function. Each

analysis involved six chickens.

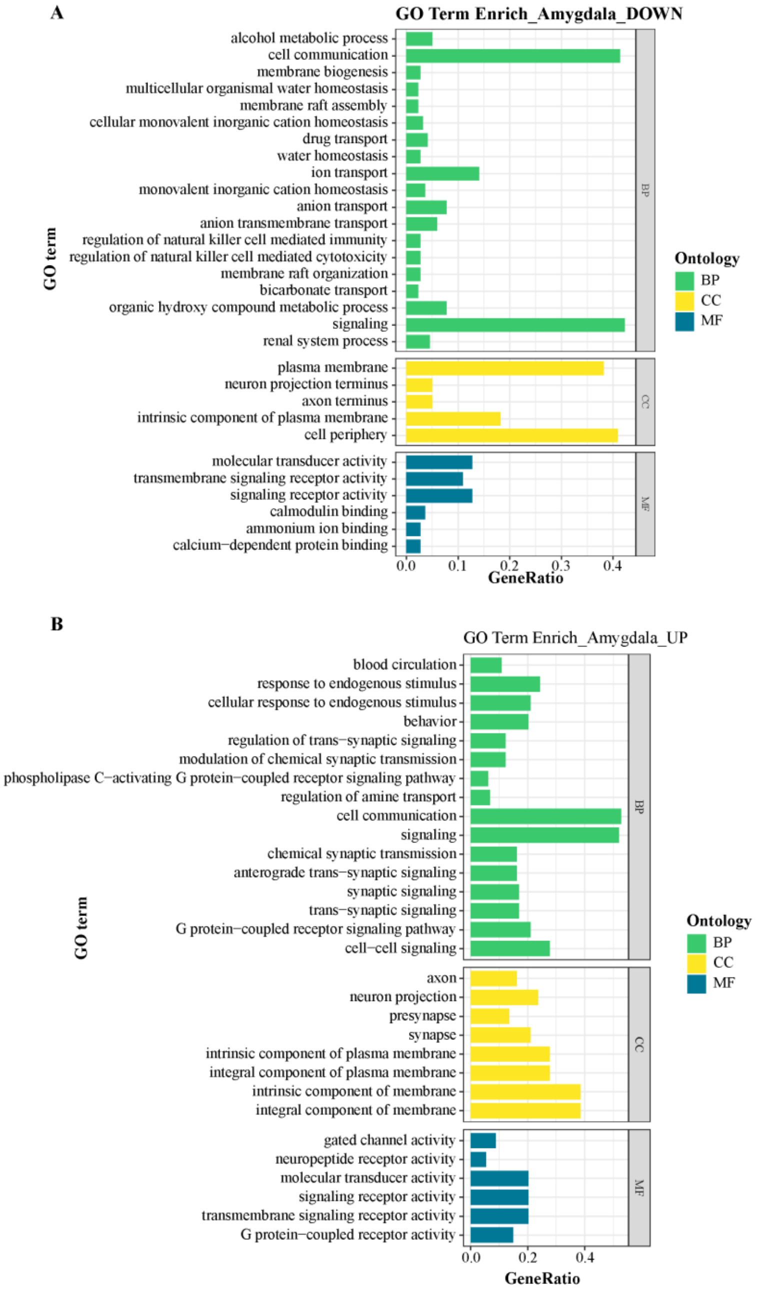

Figure S16. The top 30 enriched GO terms for significantly downregulated (**A**) and upregulated (**B**) genes in the amygdalae of feather pecking chickens. BP, biological process; CC, cellular component; MF, molecular function. Each analysis involved six chickens.

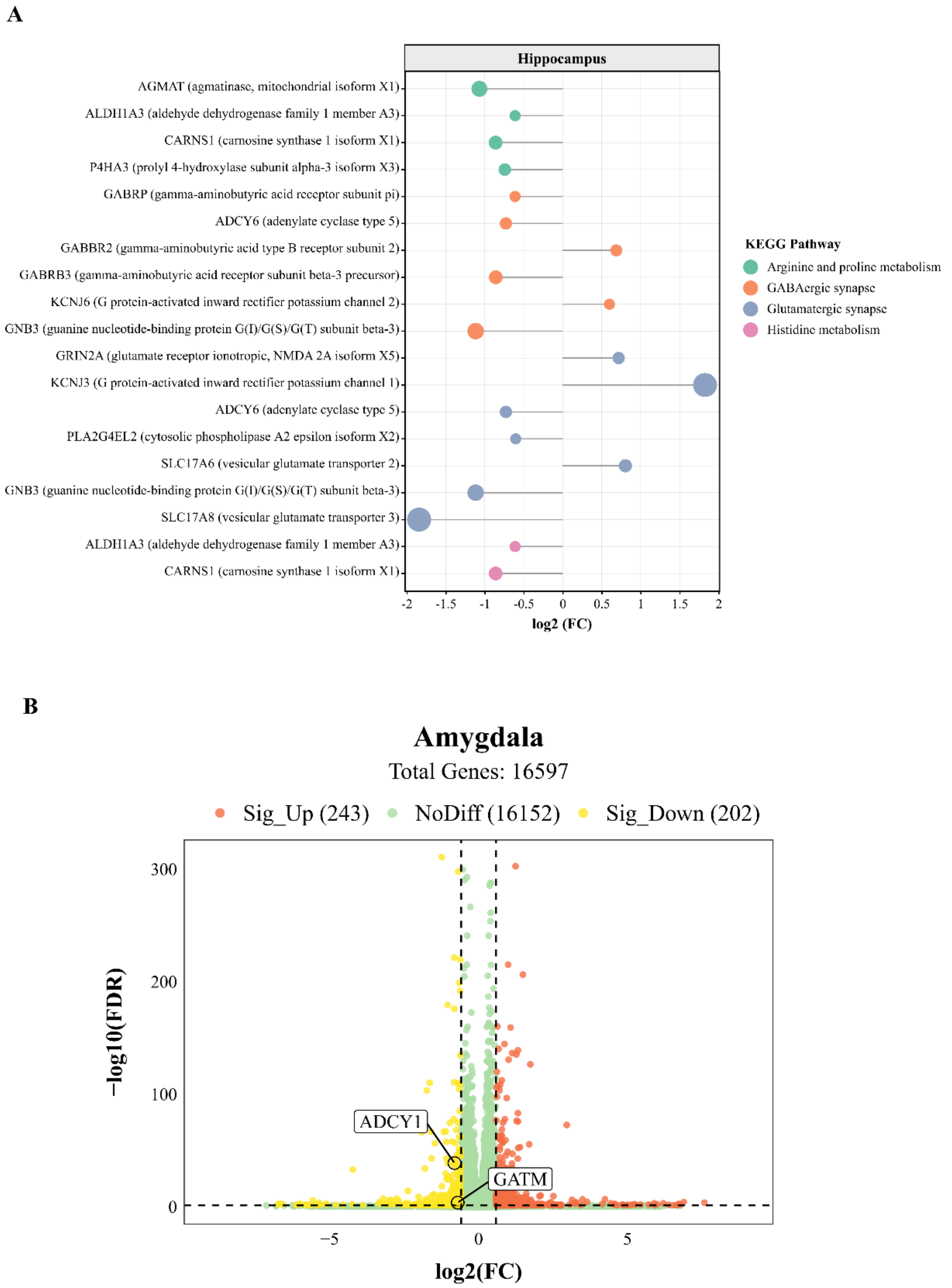

Figure S17. (**A**) Bubble mapping of the enriched GO terms in the hippocampi. (**B**) Volcano plot of differentially accumulated genes in the amygdalae. Each analysis involved six chickens. Permission was obtained from Kanehisa Laboratories to use the KEGG pathway database[1].

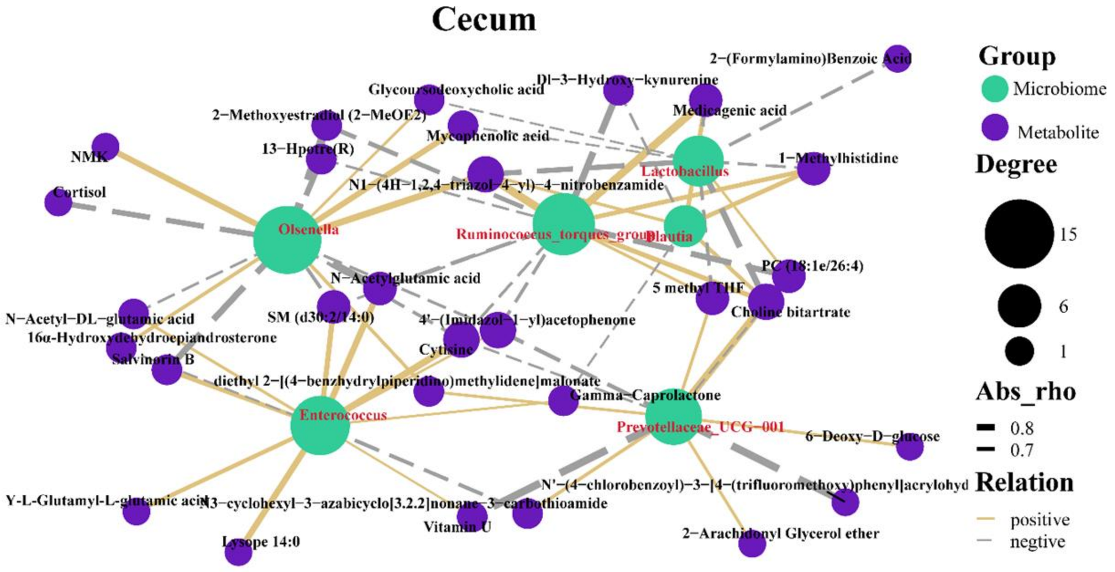

Figure S18. The correlation network of cecum microbiota and metabolites.

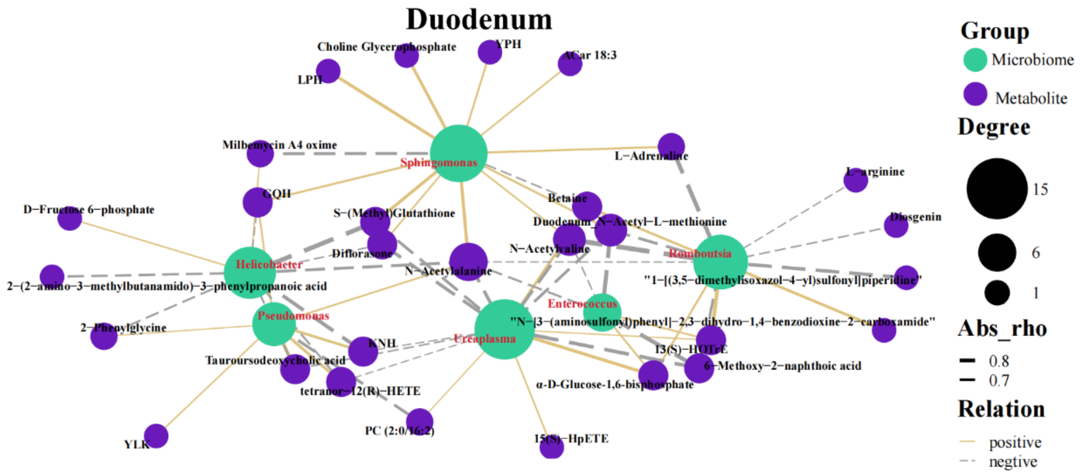

Figure S19. The correlation network of duodenum microbiota and metabolites.

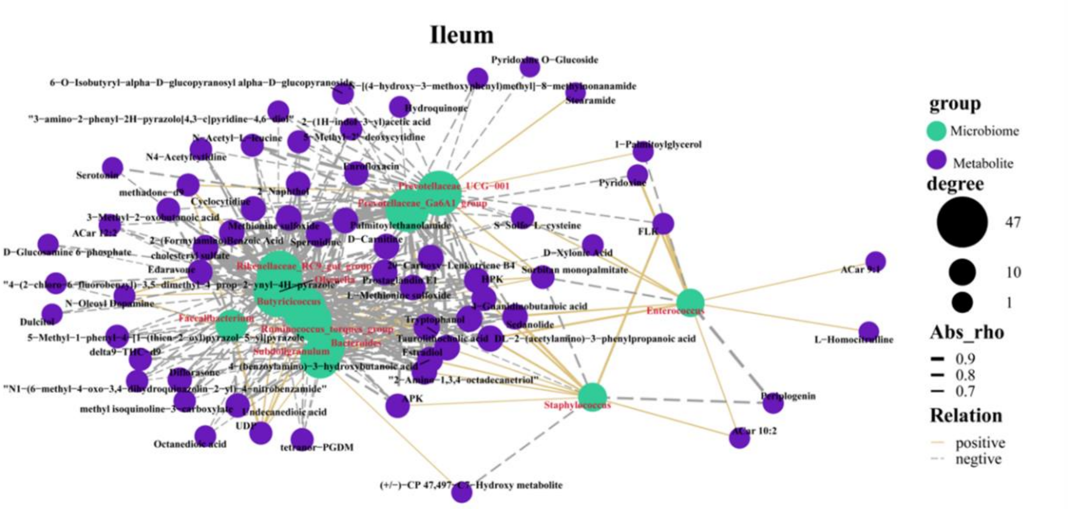

Figure S20. The correlation network of ileum microbiota and metabolites.

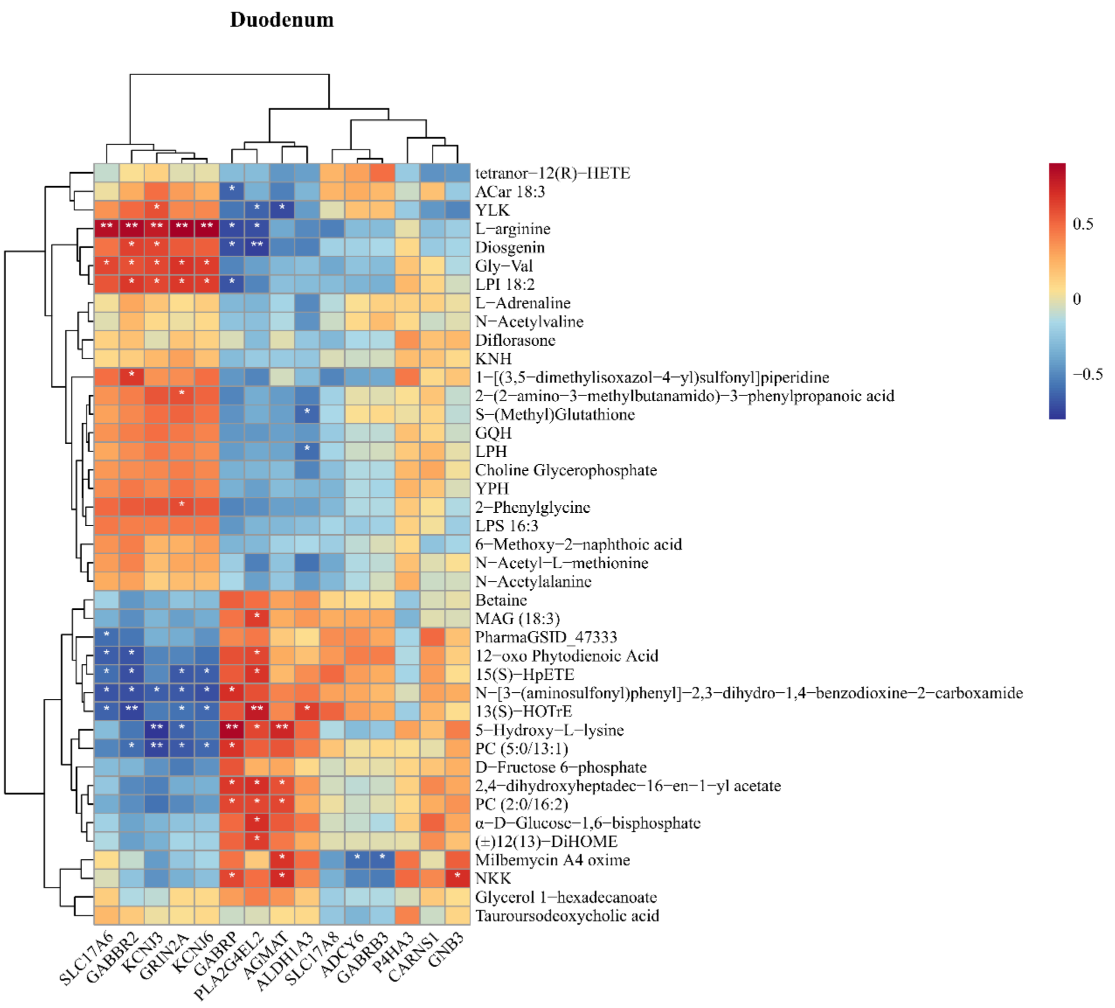

Figure S21. Correlation analysis of intestinal differential metabolites and hippocampal differential genes. Spearman correlation coefficients between the hippocampal differential genes and the differential metabolites in the duodenum. * $P < 0.05$, ** $P < 0.01$, *** $P < 0.001$.

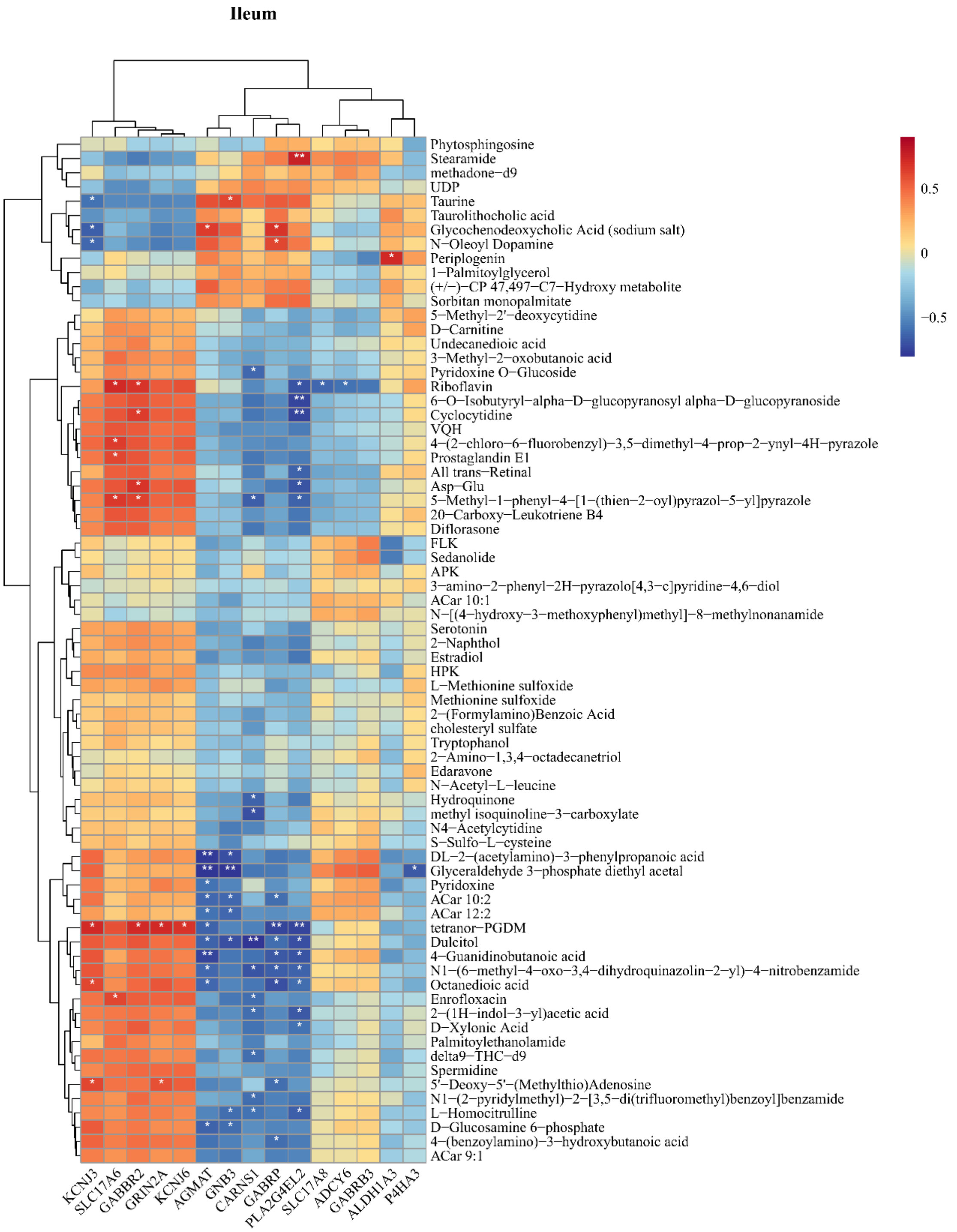

Figure S22. Correlation analysis of intestinal differential metabolites and hippocampal differential genes. Spearman correlation coefficients between the hippocampal differential genes and the differential metabolites in the ileum. * $P < 0.05$, ** $P < 0.01$, *** $P < 0.001$.

Reference:

1. Kanehisa, M.; Goto, S. KEGG: Kyoto Encyclopedia of Genes and Genomes. *Nucleic Acids Res.* **2000**, *28*, 27–30.
